# Potential Blood Biomarkers Predicting Treatment Response in Psoriasis: A Systematic Review and Meta-Analysis

**DOI:** 10.3390/jcm15156096

**Published:** 2026-08-05

**Authors:** Jose Maria Villa-Gonzalez, Irene Arevalo-Ortega, Maria Rosario Gonzalez-Hermosa, Salvador Gonzalez, Sarem Rashid, Hensin Tsao, Rosa Izu-Belloso

**Affiliations:** 1 Department of Dermatology, Cruces University Hospital, 48903 Barakaldo, Spain; 2 Wellman Center for Photomedicine, Massachusetts General Hospital, Boston, MA 02114, USA; 3 Department of Dermatology, Ophthalmology and Otorhinolaryngology, University of the Basque Country, 48940 Leioa, Spain; 4 Department of Dermatology, Basurto University Hospital, 48013 Bilbao, Spain; 5 Department of Medicine and Medical Specialties, Alcala University, 28805 Madrid, Spain; 6 Boston University School of Medicine, Boston, MA 02118, USA; 7 Department of Dermatology, Harvard Medical School, Boston, MA 02114, USA; 8 Department of Dermatology, Mass General Brigham, Boston, MA 02114, USA; 9 Biobizkaia Health Research Institute, 48903 Barakaldo, Spain

**Keywords:** psoriasis, biomarkers, treatment response, IL-23/Th17 axis, Interleukin-17, β-defensin-2, systematic review, meta-analysis

## Abstract

**Highlights:**

**Why Was the Study Undertaken?**

- Treatment response in psoriasis remains variable despite multiple systemic therapies, and patients often require sequential switching. This systematic review and meta-analysis was undertaken to evaluate whether circulating blood-derived biomarkers can predict response to systemic psoriasis treatments and support more personalized treatment selection.

**What Does This Study Add?**

- The study identifies recurrent exploratory signals for downstream IL-23/Th17 path-way biomarkers, particularly BD-2, IL-17A, and IL-17F, while upstream IL-23 showed only a non-significant trend. BD-2 showed the largest pooled statistical signal, but these findings remain hypothesis-generating rather than validated predictors.

**What Are the Implications of This Study for Disease Understanding and/or Clinical Care?**

- The findings are compatible with the hypothesis that amplified downstream pathway activity may better reflect treatment-responsive inflammation than isolated upstream cytokines. However, this interpretation remains exploratory, and no soluble circulating biomarker is currently sufficiently validated for routine treatment selection; prospective multicenter studies using standardized assays and biomarker panels are needed before clinical implementation.

**Abstract:**

**Background/Objectives:** Psoriasis is a chronic immune-mediated inflammatory disease primarily driven by the interleukin (IL)-23/T helper 17 (Th17) pathway. Despite multiple systemic therapies, treatment response varies considerably, and many patients require sequential switching. We aimed to evaluate circulating blood-derived biomarkers associated with response to systemic psoriasis therapies. **Methods:** MEDLINE and Web of Science were searched on 8 August 2025, following PRISMA guidance. The protocol was registered in PROSPERO (CRD420251129182). Eligible studies were original English- or Spanish-language studies published within the previous 10 years that evaluated circulating blood-derived biomarkers associated with cutaneous response to approved systemic psoriasis therapies in patients with psoriasis, with or without psoriatic arthritis. Genetic predictors, therapeutic drug monitoring studies, reviews, editorials, letters, conference abstracts, case reports, and non-original publications were excluded. Risk of bias was assessed using QUIPS and PROBAST. Quantitative synthesis included standardized mean difference meta-analysis and exploratory pooled *p*-value analysis. **Results:** Twenty-six studies were included. Most evaluated biologic therapies targeting TNF, IL-17, or IL-23; fewer assessed apremilast or methotrexate. Response definitions and follow-up varied across studies. Most studies showed moderate to high risk of bias, mainly due to small sample sizes, exploratory or post hoc analyses, heterogeneous treatment groups, limited confounding adjustment, multiple testing, and lack of external validation. Random-effects meta-analysis of baseline cytokines from two studies, including 71 patients per biomarker, showed no statistically significant differences between responders and non-responders for IL-17A, TNF-α, IL-6, IL-12, or IL-23. Exploratory pooled *p*-value analyses showed recurrent signals for downstream IL-23/Th17 biomarkers, particularly β-defensin-2 (BD-2; z = 4.63, *p* < 0.001), IL-17A (z = 3.27, *p* = 0.001), and IL-17F (z = 2.92, *p* = 0.003). **Conclusions:** Downstream IL-23/Th17 biomarkers, particularly BD-2 and IL-17 isoforms, showed recurrent exploratory associations with systemic treatment response. However, these findings should not be interpreted as evidence of superior predictive performance over upstream biomarkers, and no soluble circulating biomarker is currently ready for routine treatment selection.

## 1. Introduction

Psoriasis is a chronic inflammatory skin disease with multiple clinical phenotypes, including plaque psoriasis, guttate psoriasis, pustular psoriasis, and erythrodermic psoriasis, any of which may be associated with psoriatic arthritis [1]. Plaque psoriasis is the most common form, and its severity is routinely assessed using the Psoriasis Area and Severity Index (PASI), even though other indexes such as Investigator’s Global Assessment (IGA) are also used. Symptoms such as pruritus, scaling, and fissuring, together with the psychosocial burden caused by lesions in visible or sensitive body sites, contribute to the high morbidity of psoriasis and to a substantial impairment in patients’ quality of life [1,2].

**Figure 1:**
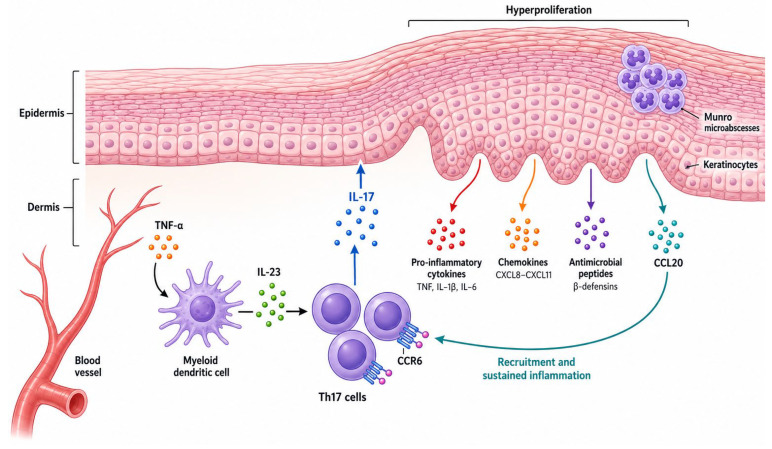
Pathogenesis of psoriasis: the IL-23/Th17 immune axis across skin layers. TNF-α activates myeloid dendritic cells, promoting IL-23 release and maintenance of Th17 cell activity through the JAK-STAT pathway. Th17-derived IL-17 acts on keratinocytes, inducing epidermal hyperproliferation and release of pro-inflammatory cytokines, chemokines, antimicrobial peptides, and CCL20. CCL20 recruits CCR6-expressing Th17 cells, reinforcing the inflammatory loop, while chemokine-mediated neutrophil recruitment contributes to Munro microabscess formation. Figure created by the authors as an original schematic representation; artificial intelligence tools were used only to support visual drafting and stylistic refinement, followed by substantive author revision and scientific verification. CCL20, C-C motif chemokine ligand 20; CCR6, C-C chemokine receptor type 6; CXCL, C-X-C motif chemokine ligand; IL, interleukin; JAK-STAT, Janus kinase-signal transducer and activator of transcription; Th17, T helper 17 cell; TNF-α, tumor necrosis factor alpha.

Psoriasis is primarily driven by the IL-23/T helper 17 (Th17) immune axis (Figure 1) [3,4]. In this pathway, inflammatory stimuli activate myeloid dendritic cells and promote the production of upstream cytokines, including IL-23 and TNF-α. IL-23 maintains pathogenic Th17 cell activity, leading to the production of downstream effector cytokines such as IL-17A, IL-17F, IL-22, and related inflammatory mediators. These cytokines act mainly on keratinocytes, inducing epidermal hyperproliferation and the release of chemokines, antimicrobial peptides, and additional pro-inflammatory mediators that sustain cutaneous inflammation [1,3,4].

Among these downstream products, β-defensins, chemokines such as CCL20, and other keratinocyte-derived molecules may reflect the amplified inflammatory response occurring in psoriatic skin. This is relevant for biomarker research because circulating upstream cytokines may not always capture local tissue inflammation, whereas downstream mediators may provide indirect evidence of pathway activation. Additional pathways, including NF-κB, IL-36/IL-1 signaling, and JAK-STAT signaling, also contribute to psoriasis pathogenesis, but the IL-23/Th17 axis remains the main therapeutic and biomarker-relevant framework for this review [1,3,4].

A wide range of systemic therapies is available for moderate-to-severe psoriasis, including conventional agents, small molecules, and biologic therapies targeting TNF, IL-17, IL-23, and IL-12/23 pathways. These treatments have substantially improved disease control, but therapeutic response remains variable across patients.

Despite the availability of multiple therapeutic options, treatment response remains highly variable among patients. A substantial proportion of individuals fail to achieve or maintain adequate disease control with initial therapy, often requiring sequential treatment changes to identify an effective option [5,6]. Because validated biomarkers are not yet routinely available to guide treatment selection, therapeutic decisions in psoriasis often remain largely empirical. This approach may contribute to delayed treatment optimization, persistent disease activity, cumulative treatment burden, unnecessary exposure to ineffective therapies, and increased healthcare costs [1,2]. Consequently, there is a growing need for strategies capable of predicting treatment effectiveness and guiding individualized therapeutic decision-making. The identification of clinical, biological, or other predictive factors of response may facilitate earlier selection of the most appropriate therapy, improve patient outcomes, avoid unnecessary adverse events, and optimize resource utilization. Therefore, this systematic review and meta-analysis aims to identify candidate biomarkers capable of predicting therapeutic response to different antipsoriatic treatments.

## 2. Material and Methods

A comprehensive literature search was conducted in the MEDLINE and Web of Science databases on 8 August 2025. The methodology followed the Preferred Reporting Items for Systematic Reviews and Meta-Analyses (PRISMA) guidelines, and the review protocol was registered in PROSPERO (International Prospective Register of Systematic Reviews) under the ID CRD420251129182.

Inclusion criteria were limited to original studies published within the last 10 years that investigated circulating blood-derived biomarkers associated with the prediction of cutaneous response to systemic therapies approved for cutaneous psoriasis in patients with psoriasis with or without psoriatic arthritis. Eligible studies included prospective or retrospective cohorts and clinical trials, provided they reported an association between one or more circulating biomarkers and treatment response.

The authors excluded studies evaluating genetic predictors, including HLA alleles or single-nucleotide polymorphisms, because these do not represent circulating biomarkers. Therefore, conclusions regarding clinical applicability apply only to soluble circulating blood-derived biomarkers and not to genomic predictors. Thus, although HLA-C*06:02 is one of the best-validated predictors of biologic response in psoriasis, including in large studies and meta-analyses, it was outside the scope of this review. The authors also excluded studies focused on therapeutic drug monitoring, including serum drug concentrations and anti-drug antibodies, because these reflect pharmacokinetic or immunogenicity monitoring rather than baseline circulating disease biomarkers.

Articles were included if they were published in English or Spanish. Reviews, editorials, letters, conference abstracts, and other non-original publications were excluded.

Data extraction was performed for each included study and organized into structured tables. Extracted data included study design, sample size, patient demographics (age and sex), type of systemic therapy evaluated, type and measurement method of biomarker(s), and main findings regarding treatment response.

Studies that included mixed populations (e.g., patients with other diseases or healthy controls) were only included if data pertaining specifically to patients with psoriasis and/or psoriatic arthritis could be extracted independently (included in the synthesis). Similarly, in studies with mixed treatment modalities, only those reporting data specific to systemic therapy were included in the synthesis.

Where information was missing or not reported, it was recorded as ‘Not available’.

### 2.1. Study Selection

An initial screening of titles and abstracts was performed. Full texts of selected studies were independently assessed for eligibility by two authors (J.V.G. and I.A.O.). Any disagreements were resolved through discussion with a third reviewer (M.I.B.).

### 2.2. Risk of Bias Assessment

Risk of bias was assessed independently at the study level according to the methodological design and objective of each article. Studies evaluating associations between circulating biomarkers and treatment response, disease activity, relapse, or treatment persistence were assessed using the Quality in Prognosis Studies (QUIPS) tool, which evaluates six domains: study participation, study attrition, prognostic factor measurement, outcome measurement, study confounding, and statistical analysis and reporting [7]. Studies that developed, tested, or proposed predictive models, biomarker-based classifiers, cut-offs, or treatment-selection algorithms were assessed using the Prediction model Risk Of Bias ASsessment Tool (PROBAST), including the domains of participants and data sources, predictors, outcome, and analysis [8]. Each domain was rated as low, moderate/unclear, or high risk of bias, as appropriate for the tool. Overall risk-of-bias judgements were based on the most concerning domains, with particular emphasis on confounding, sample size, missing data, outcome definition, multiplicity, overfitting, internal or external validation, and calibration where applicable.

### 2.3. Meta-Analysis

For studies reporting continuous biomarker levels as medians with measures of dispersion, means and standard deviations were estimated using established methods. When medians and interquartile ranges were available, mean and standard deviation were estimated. When medians with minimum and maximum values were reported, estimates were derived following the method proposed by Wan et al. [9]. For each study, biomarker levels were compared between responders and non-responders, and standardized mean differences (SMDs) were calculated using Hedges’ g. To facilitate a unified quantitative synthesis in JASP, effect sizes (including those derived from *p*-values or Odds Ratios) were converted into correlation coefficients (r). For studies reporting odds ratios (ORs) with 95% confidence intervals but no *p*-values, approximate two-sided *p*-values were estimated from the published logistic regression-derived ORs and confidence intervals using a Wald approximation on the log(OR) scale before conversion. Meta-analyses were performed using the generic inverse-variance method. To normalize the sampling distribution and stabilize the variance, correlation coefficients were transformed to Fisher’s z scale. A Random Effects model (Restricted Maximum Likelihood estimator) was applied to account for potential between-study heterogeneity. The direction of the effect was verified for each study by inspecting reported medians or Odds Ratios. To maximize statistical power given the limited number of studies, an exploratory analysis pooled studies reporting PASI75, PASI90, or PASI100 to evaluate predictors of “good clinical response” (≥PASI75), acknowledging the clinical heterogeneity of this approach. Duplicate cohorts were excluded, prioritizing the most inclusive dataset or the primary endpoint (e.g., PASI 75).

Given the limited availability of homogeneous data, a pre-specified exploratory pooling strategy was applied in cases where subgroup stratification was not feasible. Studies using different but clinically relevant response definitions (PASI75, PASI90, or PASI100), varying assessment timepoints (ranging from week 12 to week 100), and distinct systemic treatment classes (TNF, IL-17, IL-23, and TYK2 inhibitors) were combined in a single pooled analysis. Given the limited number of studies reporting comparable quantitative data, the authors performed an exploratory pooled *p*-value synthesis to identify biomarkers that showed repeated signals of association across the available literature. This analysis combined studies with different therapeutic classes, response definitions, and assessment timepoints; therefore, it was not intended to estimate a clinically interpretable pooled effect size or to support treatment-specific prediction. The resulting z-scores should be interpreted only as indicators of whether a biomarker showed recurrent statistical evidence of association across heterogeneous studies. Because the direction, magnitude, and clinical meaning of biomarker-response associations may differ according to drug mechanism, PASI threshold, and timing of assessment, these pooled results were considered hypothesis-generating and were not used to establish clinically actionable predictors. Formal subgroup analyses and direct drug-class comparisons were not conducted, given the differences in study design, patient populations, response endpoints, and assessment timepoints across included studies. All pooled analyses should therefore be interpreted as exploratory and hypothesis-generating, aimed at identifying consistent biomarker signals rather than establishing clinically actionable predictors. Importantly, Stouffer’s z-scores were used as a screening measure of combined statistical evidence and were not interpreted as estimates of effect size, biomarker discrimination, or comparative predictive performance across biomarkers or therapeutic classes.

All analyses were performed using JASP [version 0.95.4 (Apple Silicon); JASP Team 2025].

## 3. Results

**Figure 2:**
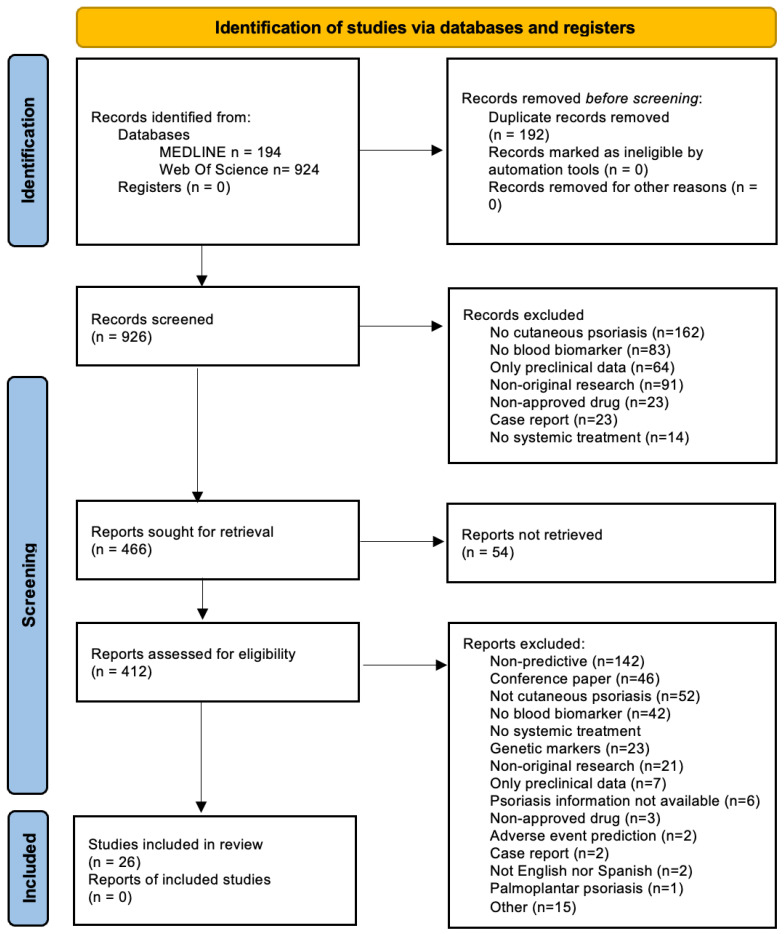
The PRISMA flowchart shows the manuscript selection process.

An initial total of 1118 records were identified through the database searches. After the removal of duplicates, 926 records remained for title and abstract screening. Of these, 466 were selected for retrieval, and 412 full-text articles were assessed for eligibility. Ultimately, 26 studies met the inclusion criteria and were included in the final analysis (Figure 2).

Thirteen (50%) studies included patients treated with TNFi, 9 (34.6%) with IL-17 inhibitors, and 8 (30.8%) with IL-23 inhibitors; 2 (7.7%) with apremilast, and 2 (7.7%) with methotrexate.

Most frequently studied biomarkers clustered around the IL-23/Th17 axis, including upstream biomarkers (IL-23), and downstream biomarkers (IL-17A, IL-17F, IL-22, TNF-α, or BD-2). Other studied markers included systemic inflammatory biomarkers (e.g., IL-6, IL-12, IL-21, IFN-α/γ, C-reactive protein (CRP), collagen-related biomarkers, vitamin D, serum amyloid A (SAA), serum calprotectin), and cellular biomarkers/immunophenotype, among others (Table 1).

The included studies evaluated treatment response at varying time points, ranging from 4 to 100 weeks after initiation. Additionally, the criteria for response differed across studies, including PASI50, PASI75, PASI90, and PASI100.

### 3.1. Risk of Bias

**Figure 3:**
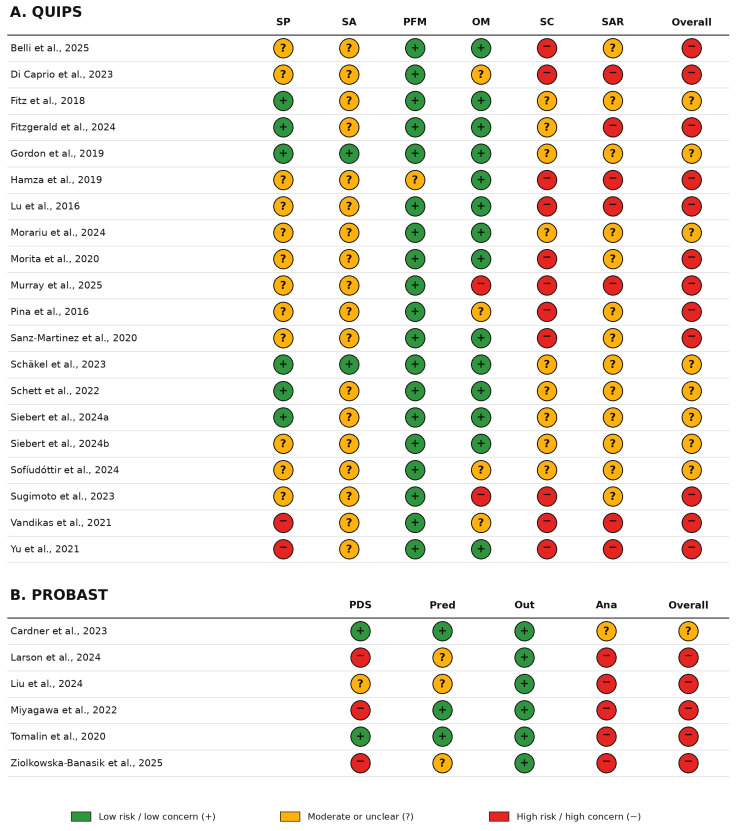
Risk-of-bias traffic-light plot across included studies. Traffic-light summary of the risk-of-bias assessment for studies evaluating circulating biomarkers as prognostic or predictive factors in psoriasis and psoriatic arthritis. Panel A shows studies assessed using QUIPS: Belli et al. (2025) [28]; Di Caprio et al. (2023) [27]; Fitz et al. (2018) [30]; Fitzgerald et al. (2024) [10]; Gordon et al. (2019) [19]; Hamza et al. (2019) [18]; Lu et al. (2016) [17]; Morariu et al. (2024) [20]; Morita et al. (2020) [15]; Murray et al. (2025) [22]; Pina et al. (2016) [29]; Sanz-Martinez et al. (2020) [25]; Schäkel et al. (2023) [11]; Schett et al. (2022) [31]; Siebert et al. (2024a) [12]; Siebert et al. (2024b) [14]; Sofíudóttir et al. (2024) [32]; Sugimoto et al. (2023) [21]; Vandikas et al. (2021) [33]; and Yu et al. (2021) [23]. Panel B shows studies assessed using PROBAST: Cardner et al. (2023) [34]; Larson et al. (2024) [26]; Liu et al. (2024) [24]; Miyagawa et al. (2022) [35]; Tomalin et al. (2020) [13]; and Ziolkowska-Banasik et al. (2025) [16]. Studies are identified within the figure by first author and publication year. Green circles with “+” indicate low risk of bias or low concern; yellow circles with “?” indicate moderate risk of bias, unclear risk, or some concerns; red circles with “−” indicate high risk of bias or high concern. Overall judgements reflect the most concerning domains, with particular attention to confounding and statistical analysis and reporting for QUIPS, and sample size, overfitting, missing data, validation, and calibration for PROBAST. Ana: analysis; OM: outcome measurement; Out: outcome; PDS: participants and data sources; PFM: prognostic factor measurement; Pred: predictors; PROBAST: Prediction model Risk Of Bias ASsessment Tool; QUIPS: Quality In Prognosis Studies; ROB: risk of bias; SA: study attrition; SAR: statistical analysis and reporting; SC: study confounding; SP: study participation.

Overall, most studies showed moderate to high risk of bias, largely reflecting exploratory biomarker analyses, small sample sizes, heterogeneous treatment groups, limited adjustment for confounding, multiple biomarker testing, and lack of external validation (Figure 3, Supplementary Table S1). Among QUIPS-assessed studies, the lowest risk of bias was observed in biomarker analyses nested within large randomized or phase III/IIIb clinical trials, particularly those evaluating guselkumab or secukinumab-associated biomarkers, whereas small single-centre observational studies and retrospective treatment-persistence analyses were generally judged at high risk. Among PROBAST-assessed studies, most prediction-model studies were considered at high or unclear risk of bias, mainly because of limited events per predictor, high-dimensional biomarker modelling, internally derived cut-offs, incomplete reporting of calibration, and absence of independent external validation. The strongest methodological support was identified for the BD-2/secukinumab analysis in psoriatic arthritis, which used large trial datasets and validation steps, although residual concerns remained due to its post hoc modelling nature. Overall, the available evidence supports several promising candidate biomarkers but indicates that most remain hypothesis-generating rather than ready for routine clinical decision-making.

### 3.2. Meta-Analysis

**Figure 4:**
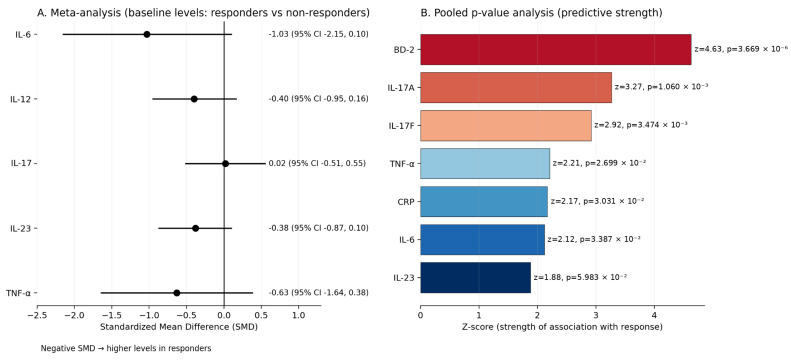
Baseline circulating biomarkers and their association with treatment response in psoriasis. (**A**) Forest plot showing standardized mean differences (SMDs) in baseline biomarker levels between responders and non−responders to systemic therapy. Data are derived from two studies evaluating IL−6, IL−12, IL−17A, IL−23, and TNF−α. Negative SMD values indicate higher baseline levels in responders. No statistically significant differences were observed, as all confidence intervals crossed the null value. Included studies comprised patients receiving etanercept and secukinumab [16,17]. (**B**) Pooled *p*−value analysis of circulating biomarkers associated with treatment response. In the exploratory pooled *p*−value analysis, downstream effectors of the IL−23/Th17 pathway showed recurrent statistical signals, particularly BD−2 (z = 4.63, *p* < 0.001), IL−17A (z = 3.27, *p* = 0.001), and IL−17F (z = 2.92, *p* = 0.003). Systemic inflammatory markers, including CRP, TNF−α, and IL−6, also showed statistically significant pooled signals (*p* < 0.05), whereas IL−23 showed a non−significant trend (z = 1.88, *p* = 0.060). These z−scores reflect pooled statistical evidence across heterogeneous studies and should not be interpreted as effect−size estimates, treatment−specific predictive estimates, evidence of superiority of downstream over upstream biomarkers, or a validated quantitative ranking of biomarker performance. Included studies comprised patients receiving different systemic therapies, including guselkumab, deucravacitinib, etanercept, and secukinumab [10,12,16,17]. BD−2: β−defensin−2; CI: confidence interval; CRP: C−reactive protein; IL: interleukin; SMD: standardized mean difference; TNF−α: tumor necrosis factor alpha; Th17: T helper 17; z: z−score.

Meta-analyses of baseline circulating levels for IL-17A, TNF, IL-6, IL-12, and IL-23 were performed based on two studies (pg/mL) (Figure 4) [16,17]. Included studies comprised patients receiving etanercept and secukinumab. The total number of meta-analyzed patients is 71 for each of the biomarkers: IL-17A, TNF, IL-6, IL-12, and IL-23. The pooled analyses revealed no statistically significant differences between responders and non-responders to biologics across the examined biomarkers. Importantly, these findings are based on a limited number of studies and a relatively small, pooled sample size, which substantially reduces statistical power. Therefore, the absence of statistically significant differences should not be interpreted as evidence of absence of association, but rather as an indication of insufficient power to detect potentially meaningful effects.

A trend toward higher levels in responders was observed for IL-6 (SMD −1.03, 95% CI −2.15 to 0.10; *p* = 0.074) and, to a lesser extent, for IL-23 (SMD −0.38; *p* = 0.12), though neither reached statistical significance. TNF and IL-12 also showed non-significant elevations in responders (SMD −0.63 and −0.40, respectively), while IL-17A showed virtually no difference (SMD 0.02).

Regarding *p*-value combination, the meta-analysis included a total of 327 patients for the assessment of IL-17A, 284 for BD-2 and CRP, 218 for IL-6 and TNF-α, 147 for IL-17F, and 71 patients for the analysis of IL-23 [10,12,16,17]. Included studies comprised patients receiving different systemic therapies, including guselkumab, deucravacitinib, etanercept, and secukinumab. The exploratory pooled *p*-value synthesis showed no statistically significant heterogeneity for the analyzed biomarkers (all heterogeneity *p*-values > 0.05). However, given the small number of contributing studies per biomarker and the heterogeneity in treatment class, response definition, and assessment timepoint, absence of statistically significant heterogeneity should not be interpreted as evidence of true clinical or biological homogeneity. In this exploratory pooled *p*-value synthesis, downstream IL-23/Th17-related markers showed recurrent statistical signals across the heterogeneous included studies, particularly BD-2 (z = 4.63, *p* < 0.001), IL-17A (z = 3.27, *p* = 0.001), and IL-17F (z = 2.92, *p* = 0.003). However, because the synthesis combined different treatment mechanisms, PASI response thresholds, and assessment timepoints, these z-scores should not be interpreted as comparable estimates of predictive strength or as evidence that these biomarkers have equivalent meaning across therapies. Rather, they indicate that these markers were repeatedly associated with response-related outcomes in the available literature. Systemic inflammatory markers (CRP, TNF-α, and IL-6) also showed statistically significant pooled signals (*p* < 0.05). However, because the pooled *p*-value synthesis does not estimate comparable effect sizes across biomarkers, these results should not be interpreted as demonstrating that systemic inflammatory markers have smaller effects than IL-23/Th17-related biomarkers. Conversely, the upstream cytokine IL-23 exhibited a clear trend towards elevation in non-responders (z = 1.88) but failed to reach formal statistical significance (*p* = 0.060) in the pooled analysis, although this finding should be interpreted cautiously given the limited sample size, potential variability across studies, and the exploratory nature of the pooled analysis.

## 4. Discussion

Despite the wide availability of systemic treatments for psoriasis, patients often undergo multiple therapies before achieving an effective response [5,6]. Therefore, there is a clear need for predictive biomarkers to enable a personalized approach to disease management, thereby avoiding unnecessary adverse events and inefficient use of healthcare resources.

This systematic review included 26 studies evaluating the ability of blood biomarkers to predict response to systemic treatments in plaque-type psoriasis.

### 4.1. Upstream Biomarkers

Considering the studies analyzing IL-23, none of them found an association between this IL and achievement of response to TNFi, IL-23 or IL-17 inhibitors [17,19,35]. One possible explanation for the lack of consistent association observed for IL-23 is the compartmentalized nature of psoriatic inflammation. Psoriasis is primarily a skin-driven disease, and circulating levels of upstream cytokines may not fully reflect local immune activity within lesional tissue. In contrast, downstream effectors of the IL-23/Th17 axis, such as IL-17 isoforms and antimicrobial peptides like BD-2, may represent amplified outputs of the inflammatory cascade and may therefore be more readily detectable in peripheral blood. However, this remains a biologically plausible interpretation rather than direct evidence that downstream biomarkers are intrinsically superior to upstream cytokines. The available data only support the hypothesis that downstream markers may capture treatment-responsive inflammation in some settings, and this hypothesis requires prospective validation.

### 4.2. Downstream Biomarkers

IL-17 functions as the key downstream effector cytokine of the IL-23/Th17 axis. Several studies reported associations between specific IL-17 isoforms and treatment response; however, these significant findings were generally confined to particular patient subgroups rather than being consistently observed across entire study cohorts. For example, Schäkel et al. found that baseline IL-17A was higher in non-responders to guselkumab (PASI > 0 at weeks 20 and 28) compared to responders, but only in those with a disease lasting > 2 years [11]. Similarly, Siebert et al. described that those who achieved PASI75 and PASI 90 response at week 24 with guselkumab had significantly higher baseline IL-17F levels compared with non-responders (*p* = 0.018 and *p* = 0.008 respectively), but only in the biologic-naive patient group [12]. In the group of inadequate responders to TNFi, a higher level of IL-17A was observed for responders (*p* = 0.035). These findings indicate a potential trend toward improved response in selected patient subgroups with elevated baseline IL-17 subunit levels, although this pattern was not consistently observed across all cohorts or studies.

Despite being different subunits, these discrepancies with Schäkel et al. for IL-17 may be due to the subgroup analysis that is performed in this study, considering that when all patients (short- and long-lasting disease) were analyzed together no differences were found [11]. Siebert et al. did not report results of predictive capacity for the whole cohort (biologic-naive or inadequate responders to TNFi patients), and thus, there might be a selection bias [12].

Overall, these studies suggest a possible association between higher baseline IL-17-related cytokines and treatment response in selected contexts.

Similarly, in another study by Siebert et al., the authors found that early changes in IL-17A and IL-17F showed moderate positive correlations with PASI improvement at week 100, under treatment with guselkumab [14].

Deucravacitinib was the only treatment showing a predictive effect across the overall cohort. Higher baseline IL-17A levels were observed among patients who achieved PASI75 at week 16 compared with non-responders, both in the deucravacitinib 6 mg group (responders: 0.72 vs. non-responders: 0.49 ng/L; *p* < 0.05) and in the 12 mg group (responders: 0.665 vs. non-responders: 0.39 ng/L; *p* < 0.05). When baseline biomarkers were dichotomized into high and low subgroups using the median baseline IL-17A level as cut-off (0.575 ng/L), patients receiving deucravacitinib 6 mg once daily with high baseline IL-17A levels showed significantly higher PASI75 response rates than those with low IL-17A levels (57.6% vs. 23.1%; *p* = 0.02) [10]. The association between higher baseline IL-17A and improved PASI75 response to deucravacitinib is biologically plausible, as TYK2 mediates signaling downstream of IL-23, a key cytokine involved in maintaining the Th17 phenotype and sustaining IL-17A–driven keratinocyte inflammation. Thus, elevated baseline IL-17A may identify patients with a more IL-23/Th17-dependent inflammatory endotype, in whom selective TYK2 inhibition more effectively suppresses the pathogenic pathway.

Nonetheless, there are some other studies that did not find an association for IL-17A [17,19,30,35] or IL-17F [11,19].

Taken together, IL-17-related biomarkers show a biologically plausible but inconsistent signal across studies.

Regarding IL-22, another key downstream interleukin in the IL-23/Th17 axis, there was one study that found that, in previously inadequate responders to TNFi but not biologic-naïve patients, PASI90 responders had significantly higher baseline IL-22 (*p* = 0.025) levels compared with non-responders. Siebert et al. found a correlation in early changes in IL-22 from baseline to week 24 with long-term clinical response at week 100 with guselkumab, where decreases in IL levels correlated with improved skin response [14]. However, there were some studies that did not find any association with prediction of response [11,19,35]. Considering the totality of the evidence, IL-22 does not demonstrate sufficient consistency to be regarded as a robust global predictor of treatment response.

Similarly, TNF-α, a pleiotropic cytokine involved both upstream and downstream in the inflammatory cascade, was associated with achievement of IGA 0/1 at week 24 under guselkumab treatment (*p* = 0.016), but this association was observed only in patients who were previously inadequate responders to TNF inhibitors [12]. Other studies did not show association [14,16,17,35]. Thus, overall, the available evidence does not support TNF-α as a consistent or broadly applicable predictor of treatment response.

Taken together, cytokines within the IL-23/Th17 axis show heterogeneous and often subgroup-specific associations with treatment response. Downstream effectors such as IL-17 isoforms and BD-2 emerged as biologically plausible candidate signals, but the available evidence remains insufficient to establish their comparative superiority over upstream regulators. This variability highlights the complexity of inflammatory signaling in psoriasis and suggests that single cytokine measurements may have limited predictive capacity when evaluated in isolation.

Regarding BD-2, several studies reported an association between higher baseline levels and improved response to systemic therapy, although in some cases these findings were restricted to specific patient subgroups rather than observed across the entire study population. For example, Siebert et al. found that biologic-naive patients who achieved PASI 90 showed higher baseline BD-2 levels (*p* = 0.013), and among previously inadequate responders to TNFi, PASI90 responders to guselkumab had significantly higher baseline BD-2 (*p* = 0.004) [12]. In the latter group, similarly, patients achieving an IGA 0/1 response at week 24 under guselkumab exhibited higher baseline BD-2 (*p* = 0.005) levels. In another study, Siebert et al. found that changes in BD-2, among other biomarkers mentioned above, showed moderate positive correlations with PASI improvement at week 100 with guselkumab [14]. Likewise, in the study by Morita et al., patients treated with Secukinumab showed a marked and rapid reduction in serum BD-2 levels at week 2, which preceded clinical improvement in PASI at week 16 in some of the patients [15]. Similarly, FitzGerald et al. found an association between a higher PASI improvement under deucravacitinib therapy, and higher baseline BD-2 levels [OR 15.41 (95% CI 3.36–70.71)], but this association was not found for PASI75 achievement [10]. In contrast, one study evaluating secukinumab [34] and another assessing guselkumab [11] did not identify a significant association. Notably, the latter defined response as achievement of PASI100, a particularly stringent endpoint that may have reduced its ability to detect associations, despite evaluating guselkumab as in the other studies that reported positive findings. Taken together, BD-2 appears to be one of the most promising soluble candidate biomarkers identified in this review. It showed the numerically largest exploratory pooled signal in the *p*-value screening analysis (z = 4.63, *p* < 0.001), but this should not be interpreted as evidence of the largest effect size or strongest clinical predictive performance. The study-level evidence remains mixed, with positive findings in some cohorts and no significant association in others. Therefore, BD-2 should be considered a priority candidate for future validation rather than an established predictor of treatment response.

No association was found for IFN-γ [16,35].

### 4.3. Systemic Inflammatory Biomarkers

In terms of IL-6, Siebert et al. were the only authors finding that lower baseline levels could predict IGA 0/1 achievement (*p* = 0.013), but only in biologic-naïve patients [12]. Other studies did not find any association [14,16,35].

IL-12 levels were found to be higher at baseline in PASI75 responders at week 24 under etanercept therapy in the study by Lu et al. (*p* = 0.028); however, it did not remain a statistically significant independent predictor in multivariable analysis, although a trend toward association persisted (*p* = 0.124; OR = 1.002, 95% CI 0.999–1.005) [17]. Similarly, Ziolkowska-Banasik et al. did not find any association for IL-12 in patients under secukinumab [16].

Therefore, the available data may suggest that IL-6 and IL-12 are unlikely to serve as reliable or consistent predictors of treatment response.

In addition, higher baseline IL-19 levels were also found to be significantly associated with PASI75 achievement at week 16 under deucravacitinib treatment [10].

Similarly, higher baseline IL-13 and lower baseline IL-18, IL-18/IL-13, and IL-17/IL-13 ratios were associated with complete clearance (PASI = 0) at week 12 under secukinumab therapy [16].

Overall, most systemic inflammatory biomarkers showed limited reproducibility, with several associations emerging from single cohorts only.

Regarding non-IL inflammatory biomarkers, C-reactive protein (CRP) was associated with significantly higher odds of achieving PASI75 with deucravacitinib compared with placebo in both high- and low-CRP subgroups, with ORs of 5.40 (95% CI 1.76–16.58) and 2.84 (95% CI 1.00–8.11), respectively [10]. However, because this association was observed across both CRP strata, and because CRP is a nonspecific acute-phase reactant rather than a psoriasis-specific mediator, these findings may reflect systemic inflammatory burden rather than selective activation of a psoriasis-specific pathway. In the deucravacitinib study, CRP was categorized among general inflammatory biomarkers, together with IL-6 and TNF-α, and baseline CRP showed only weak association with PASI, while inflammatory markers were more broadly associated with composite psoriatic arthritis disease activity measures^12. Therefore, CRP may capture a component of overall inflammatory burden associated with treatment responsiveness, but its nonspecific nature limits its utility as a psoriasis-specific predictive biomarker. Consistently, two other studies failed to identify a significant association between CRP and treatment response [14,17]. Thus, when considered collectively, further studies are warranted to establish whether CRP has reproducible predictive value in psoriasis treatment response or primarily reflects nonspecific systemic inflammation. Chemokines, including CCL22/MDC, have also been linked to response to guselkumab, although the direction of association was not specified [19].

It should be highlighted that baseline serum calprotectin emerged as a promising prognostic biomarker in the context of psoriasis pathogenesis. Patients achieving PASI50 at month 3 showed significantly higher baseline calprotectin levels compared with non-responders (*p* = 0.015), and receiver operating characteristic (ROC) analysis demonstrated good discriminative ability for predicting methotrexate response [18]. Given calprotectin’s central role in neutrophil-driven inflammation and innate immune activation in psoriasis, these findings support its potential clinical utility as a biologically plausible predictor of treatment response. Its clinical appeal is further strengthened by the relative availability of calprotectin assays in routine laboratory settings, which could facilitate translation into real-world practice if its predictive value is confirmed in larger, independent cohorts.

In addition, collagen-associated biomarkers, including C1M, C3M, and C6M, failed to achieve association with response under guselkumab therapy [14,31].

### 4.4. Cellular Biomarkers/Immunophenotype

Cell population-related biomarkers have all been explored for their potential to predict treatment response in psoriasis. Among cellular markers, higher baseline frequencies of CD4^+^CD41a^+^ cells and CD41a^+^ regulatory T cells were observed in PASI75 responders to adalimumab at week 24, whereas CD4^+^CD161^+^CD41a^+^ cells did not differ between groups [25]. Lower baseline CD147 expression on CD4^+^ T cells and Th17 cells was associated with greater subsequent PASI improvement under IL-17 inhibitor therapy, and early increases in CD147 after four weeks of treatment correlated positively with clinical response, with larger increases associated with greater reductions in PASI [24]. Similarly, patients with baseline monocyte doublets ≥ 0.4% demonstrated markedly better responses to apremilast, while those below this threshold showed minimal improvement; these responders also exhibited distinct gene expression profiles, including key genes (*ATL3*, *PDK4*, *PDE4A*, *PDE8A*, *ADCY4/6*, *GNAL*, *AMPD2*, *HPRT1*) that differentiated them from non-responders and correlated with both monocyte doublets and clinical outcomes [26].

Across studies, cellular immunophenotyping suggests that baseline immune cell composition may influence treatment response, particularly in pathways involving T-cell activation and regulatory mechanisms. However, methodological heterogeneity and small sample sizes currently limit the translation of these findings into clinical practice.

In another study, Yu et al. found that baseline proportions of circulating Tregs and TIGIT expression on CD4+ T cells were negatively correlated with PASI reduction at week 10 under infliximab treatment [23]. Complementing these cellular observations, humoral immune markers such as baseline and post-adalimumab κ and λ free light chain levels were significantly elevated in PASI75 responders, whereas non-responders showed no significant changes [27].

Other biomarkers such as SIRI or derived neutrophil-to-lymphocyte ratio (d-NLR) were found to be higher in responders [20]. In contrast, another study reported that SII, NLR, and PLR were elevated in non-responders, with higher baseline SII identified as a significant predictor of biologic switching in multinomial logistic regression analysis [22]. Additionally, a separate study found that patients with high pretreatment neutrophil and platelet counts, PLR, and SII had significantly lower continuation rates for conventional systemic therapies, whereas these baseline scores did not influence biologic treatment retention [21]. These discrepancies may be attributable to differences in outcome definitions: Murray et al. classified maintenance on the same biologic as a response, considering biologic switching as non-response [22], whereas Morariu et al. evaluated achievement of PASI100 at six months. In addition, results regarding d-NLR and SIRI should be interpreted with caution, as the 95% confidence intervals for PASI100 crossed unity, indicating limited precision of the estimates [20].

Taken together, routine hematologic indices may reflect systemic inflammatory burden, but their predictive value for treatment response remains inconsistent across studies and treatment modalities.

### 4.5. Metabolic Biomarkers

Metabolic markers further contribute to this multidimensional picture: lower baseline triglycerides, total cholesterol, and leptin were associated with higher PASI responses at week 16 for IL-17/IL-23-targeted therapies, while elevated baseline leptin independently predicted poorer PASI90 outcomes in patients treated with IL-23 inhibitors, with no significant associations observed in IL-17-treated cohorts [28].

These findings suggest that metabolic status may influence therapeutic outcomes, particularly for IL-23-targeted therapies, although evidence remains limited and requires confirmation in larger cohorts.

Higher baseline ADMA (asymmetric dimethylarginine) levels were significantly associated with greater reductions in BSA and PASI at 6 months, indicating that elevated baseline ADMA predicts a better clinical response to treatment [29]. In contrast, OPG (osteoprotegerin) levels did not correlate with treatment outcomes.

### 4.6. Other Biomarkers

Importantly, several recent studies suggest that single biomarkers may be insufficient to capture the complexity of treatment response in psoriasis. These findings support the development of multimarker predictive models integrating different layers of biological information, including cytokine profiles, cellular immunophenotypes, and metabolic parameters. Such multidimensional approaches, particularly when combined with machine learning techniques, may ultimately provide more robust prediction tools, although they require prospective validation before clinical application.

It should be highlighted that Tomalin et al. demonstrated that etanercept’s response is linked to a combination of inflammatory and cardiovascular biomarkers [13]. Its core signature integrates key inflammatory proteins (CCL20, CXCL10, IFNγ, and IL-12 receptor B) with cardiovascular proteins GAL and LEP, which were downregulated in patients who responded successfully, highlighting the potential value of integrated biomarker signatures combining inflammatory and cardiometabolic pathways.

Several additional biomarkers have been evaluated in isolated studies but have not demonstrated consistent associations with treatment response (Supplementary Table S1).

### 4.7. Baseline Versus Dynamic Biomarkers

Most studies included in this review evaluated biomarkers measured before treatment initiation. Baseline biomarkers are intended to identify patients who are more likely to respond before exposure to a therapy and could therefore, if adequately validated, support initial treatment selection. Their main potential advantage is that they are available at the time of the first therapeutic decision. However, baseline measurements may be affected by between-patient and between-study heterogeneity, including differences in disease duration, inflammatory burden, previous biologic exposure, patient characteristics, and biomarker measurement methods. These factors may contribute to the inconsistent and subgroup-specific findings observed across studies.

Dynamic biomarkers assess biological changes after treatment initiation and may reflect pharmacodynamic activity or an emerging biological response. Several included studies illustrate this potential. During IL-17 inhibitor therapy, lower baseline CD147 expression on CD4^+^ T and Th17 cells and a greater increase in CD147 expression at week 4 were associated with greater subsequent PASI improvement. Serum BD-2 decreased markedly as early as week 2 during secukinumab treatment, preceding PASI improvement at week 16 in some patients. During guselkumab treatment, changes in IL-17A, IL-17F, IL-22, and BD-2 from baseline to week 24 were associated with PASI improvement at week 100. In addition, baseline and post-adalimumab κ and λ free light-chain levels were higher in PASI75 responders, whereas nonresponders showed no significant changes in a small study.

These findings suggest that dynamic measurements may provide information not captured by a single baseline value because each patient serves partly as their own biological reference. Nevertheless, the reported on-treatment changes are more appropriately regarded as candidate pharmacodynamic or response biomarkers rather than pretreatment predictive biomarkers. Only measurements obtained shortly after treatment initiation, such as CD147 at week 4 or BD-2 at week 2, can reasonably be considered candidate early-response biomarkers. Unlike baseline markers, dynamic biomarkers cannot guide the initial treatment choice because exposure to the selected therapy has already occurred. Their possible clinical application would instead be the identification of biological response or lack of response after treatment initiation, potentially supporting treatment continuation, closer monitoring, or earlier reassessment. However, the optimal sampling time, magnitude of change, assay thresholds, and incremental value over clinical assessment have not been established. Direct comparisons of baseline and dynamic biomarker performance were also generally unavailable. Consequently, the current evidence does not demonstrate that dynamic biomarkers have greater predictive value than baseline biomarkers, and both approaches require prospective evaluation using standardized timepoints, predefined cut-offs, and clinically relevant decision algorithms.

### 4.8. Meta-Analysis

Despite the heterogeneity among studies, considering all those biomarkers for which data was available, a combination of the *p*-values was performed. Across the exploratory pooled *p*-value analyses, biomarkers related to downstream IL-23/Th17 pathway activity showed recurrent statistical signals, particularly BD-2 (z = 4.63, *p* < 0.001), IL-17A (z = 3.27, *p* = 0.001), and IL-17F (z = 2.92, *p* = 0.003). However, because studies differed substantially in drug mechanism, response threshold, timing of assessment, and direction of association, these findings should not be interpreted as evidence of a unified predictive hierarchy across therapies. Rather, they identify BD-2 and IL-17 isoforms as biologically plausible candidate biomarkers that warrant prospective validation. The pooled z-score for BD-2 was numerically the largest in this screening analysis (z = 4.63, *p* < 0.001), but Stouffer’s method combines statistical evidence rather than estimating effect magnitude. Therefore, this result should not be interpreted as demonstrating that BD-2 has the strongest clinical predictive performance, the largest treatment-response effect, or treatment-agnostic utility. Although the upstream regulator IL-23 showed a directional trend consistent with the other biomarkers, its lack of statistical significance (z = 1.88, *p* = 0.060) should be interpreted cautiously. This pattern could reflect limited sample size, study heterogeneity, compartmentalized biology, or differences in the detectability of circulating proximal cytokines compared with downstream pathway markers; however, the present data cannot distinguish among these explanations. Consequently, these findings support further investigation of downstream pathway markers as candidate biomarkers, but they do not establish their superiority over upstream or systemic inflammatory markers for clinical prediction. A potential explanation for this observation is related to signal amplification along the inflammatory cascade. While upstream cytokines such as IL-23 act as regulatory drivers, their circulating levels may not accurately reflect the magnitude of downstream pathway activation. In contrast, downstream effectors such as IL-17 isoforms and BD-2 represent amplified outputs of the pathway, making them more detectable in peripheral blood and more closely aligned with tissue-level inflammatory activity. This provides one possible biological interpretation for the recurrent exploratory signals observed for downstream biomarkers in the available studies, although comparative predictive performance cannot be established from the current evidence.

Similarly, a meta-analysis of baseline circulating IL-17A, TNF-α, IL-6, IL-12, and IL-23 levels, incorporating data from Lu et al. and Ziolkowska-Banasik et al., found no statistically significant differences between responders and non-responders for any of the evaluated biomarkers [16,17]. However, heterogeneity in outcome definitions and assessment time points across studies should be noted (e.g., Lu et al. [17] reported PASI75 at week 24, whereas Ziolkowska-Banasik et al. [16] reported complete clearance (PASI = 0) at week 12).

In this secondary analysis, responders tended to exhibit higher baseline IL-6 levels, although this did not reach statistical significance, with a smaller and similarly non-significant pattern observed for IL-23. TNF-α and IL-12 showed small, non-significant shifts favoring higher levels in responders, while IL-17A displayed no meaningful difference between response groups.

Taken together, these findings are consistent with the biological relevance of the IL-17 pathway in psoriasis, but they remain insufficient to define a treatment-agnostic predictive biomarker.

Notably, substantial heterogeneity was observed for IL-6 (I^2^ = 76%) and TNF-α (I^2^ = 73%). This high variability may be attributable to differences in study design and response criteria: Lu et al. defined response as PASI75 at week 24 for etanercept, whereas Ziolkowska-Banasik et al. used a stricter outcome (PASI 100) at an earlier timepoint (week 12) for secukinumab. Conversely, heterogeneity was low to negligible for IL-12 (22%), IL-17A (14%), and IL-23 (0%) [16,17]. This variability highlights the challenges of comparing biomarker performance across studies with different treatment classes, response definitions, and follow-up durations. However, these preliminary data may provide a starting point for generating hypotheses about biomarker-informed response prediction in psoriasis. Future studies integrating multiple biomarkers within pathway-based models may improve predictive performance beyond single-marker approaches.

The discrepancies between meta-analytic approaches likely reflect differences in sample size and statistical power, as the analysis restricted to two studies included a substantially smaller pooled population [16,17]. Therefore, the absence of significant associations in this subset should be interpreted cautiously.

It is also important to distinguish between predictive biomarkers, which identify patients likely to respond before treatment initiation, and pharmacodynamic biomarkers, which reflect biological response to therapy. Some of the observed associations, particularly those involving dynamic changes in cytokine levels, may fall into the latter category.

Overall, the available evidence supports further investigation of downstream pathway activity as a biologically plausible source of candidate predictive biomarkers in psoriasis, while remaining insufficient to establish their superiority over upstream or systemic inflammatory markers. However, the current data are insufficient to determine whether downstream biomarkers are clinically more informative than upstream regulatory cytokines. This concept should therefore be regarded as a testable hypothesis for future prospective studies rather than as an established conclusion.

### 4.9. Candidate Biomarkers According to Therapeutic Class

When considered according to therapeutic class, the available evidence does not identify any validated circulating biomarker capable of guiding selection among systemic treatments. For TNF inhibitors, no single biomarker showed consistent replication. Candidate signals included an integrated inflammatory and cardiometabolic protein signature associated with etanercept response, together with isolated cellular or humoral findings under infliximab or adalimumab, including Treg/TIGIT phenotypes, CD4^+^CD41a^+^ cell populations, and κ and λ free light chains. However, these associations were generally derived from individual studies and require independent validation. For IL-17 inhibitors, BD-2 and CD147 appear to be among the more notable candidates. In a small secukinumab study, a rapid reduction in BD-2 at week 2 preceded later clinical improvement in some patients, whereas lower baseline CD147 expression followed by an increase at week 4 was associated with greater subsequent PASI improvement under IL-17 inhibitor therapy. Baseline cytokine ratios also showed preliminary associations in a single small secukinumab study. A lower IL-18/IL-13 ratio yielded an AUC of 0.86, while a lower IL-17/IL-13 ratio yielded an AUC of 0.89 for complete clearance (PASI = 0) at week 12, although these findings have not been replicated. For IL-23 inhibition, BD-2 showed the most repeatedly reported candidate signal, whereas other cytokine findings were subgroup-specific and, for IL-17A, directionally inconsistent. Higher baseline IL-17A was associated with response among TNF inhibitor-inadequate responders in one study but with nonresponse among patients with disease duration longer than two years in another. Higher IL-17F or IL-22 and lower TNF-α or IL-6 were associated with response only in selected biologic-naive or TNF inhibitor-inadequate-responder subgroups. Metabolic markers were evaluated in one study, in which higher baseline leptin independently predicted poorer PASI90 response in the IL-23 inhibitor subgroup. For TYK2 inhibition with deucravacitinib, higher baseline IL-17A provided the clearest overall-cohort signal. Higher BD-2 was associated with greater PASI improvement in the combined-dose analysis but not with PASI75, whereas higher IL-19 was associated with PASI75 only in the 12 mg group. Evidence for apremilast and conventional systemic treatments was particularly limited: baseline monocyte doublets were associated with apremilast response in one study, whereas serum calprotectin was associated with methotrexate response in another. Overall, BD-2 and IL-17-related markers warrant prioritization for further investigation, particularly in treatments targeting the IL-23/Th17 pathway. Nevertheless, heterogeneity in therapies, endpoints, populations, assays, and assessment times precludes comparative ranking or clinical use for selecting among therapeutic classes.

### 4.10. Clinical Applicability

**Figure 5:**
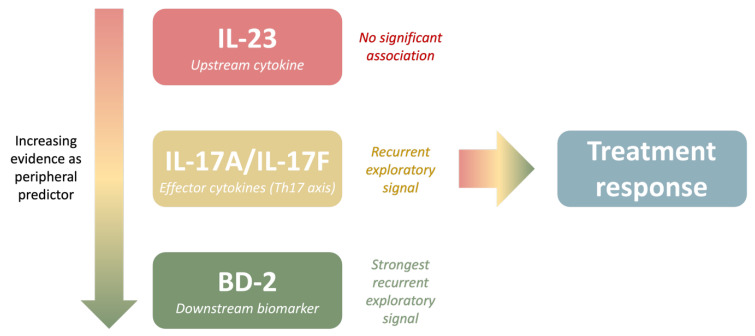
Hypothesis-generating pathway map of candidate circulating biomarkers associated with systemic treatment response in psoriasis. The figure summarizes recurrent exploratory signals identified through qualitative synthesis and pooled *p*-value screening. Downstream IL-23/Th17 pathway biomarkers, particularly BD-2 (z = 4.63, *p* < 0.001), IL-17A (z = 3.27, *p* = 0.001), and IL-17F (z = 2.92, *p* = 0.003), showed repeated statistical signals across heterogeneous studies. IL-23, an upstream regulatory cytokine, showed a non-significant trend (z = 1.88, *p* = 0.060). Biomarker placement reflects biological position within the IL-23/Th17 pathway rather than validated predictive performance. The z-scores indicate pooled statistical evidence and should not be interpreted as effect sizes, treatment-specific estimates, or quantitative rankings of clinical utility. BD-2 and IL-17 isoforms are highlighted as candidates for prospective validation, not as established predictors. BD-2, β-defensin-2; IL, interleukin; PASI, Psoriasis Area and Severity Index; Th17, T helper 17.

No soluble circulating biomarker identified in this review currently meets the analytic-validity, clinical-validity, and clinical-utility criteria required for routine psoriasis treatment selection. Although several candidates, including BD-2, IL-17A, IL-17F, calprotectin, selected cytokine ratios, metabolic markers, and cellular immunophenotypes, showed exploratory associations with response, these signals were generally derived from small cohorts, post hoc or retrospective analyses, heterogeneous treatment classes, variable endpoints, and non-standardized assays (Figure 5).

Accordingly, these biomarkers should not currently be used to select, exclude, or prioritize a specific systemic therapy outside a research setting. Their main value at present is translational: they may help define candidate pathways and inform the design of prospective biomarker-stratified studies, but they do not yet provide a basis for routine biomarker-guided prescribing.

This clinical-applicability assessment refers only to soluble circulating biomarkers; genetic predictors such as HLA-C*06:02 were outside the scope of this review and should not be conflated with the biomarker candidates evaluated here.

## 5. Conclusions

This systematic review and quantitative synthesis indicate that circulating biomarkers related to the IL-23/Th17 pathway, particularly downstream effectors such as IL-17A, IL-17F, and BD-2, showed recurrent exploratory association signals in the available literature. However, because these signals derive from heterogeneous studies differing in treatment class, response endpoint, and timing, they should be interpreted as hypothesis-generating rather than as validated predictors of systemic treatment response. Among them, BD-2 showed the numerically largest exploratory pooled z-score in the *p*-value screening analysis (z = 4.63, *p* < 0.001), supporting its prioritization for future validation. However, this should not be interpreted as evidence that BD-2 is the strongest clinical predictor, given the mixed study-level evidence, heterogeneity in endpoints and treatment classes, and the absence of validated effect-size estimates. In contrast, upstream cytokines such as IL-23 showed only marginal or inconsistent associations, including a non-significant pooled trend (z = 1.88, *p* = 0.060). These findings are compatible with the hypothesis that amplified downstream pathway activity may be informative, although this remains speculative and requires testing in adequately powered prospective studies.

Traditional systemic inflammatory markers, including CRP, IL-6, and TNF-α, displayed limited and heterogeneous predictive capacity across studies. Although these biomarkers may reflect overall inflammatory burden, they lack pathway specificity and do not consistently differentiate responders from non-responders. Additional markers, including calprotectin, selected interleukins (e.g., IL-13, IL-18, IL-19), metabolic parameters, and specific immune cell phenotypes, appear promising in certain therapeutic contexts. Similarly, beyond individual cytokine levels, ratio-based biomarkers (such as IL-18/IL-13 and IL-17/IL-13) suggest that relative pathway balance rather than absolute cytokine concentration may better capture the immunologic context associated with therapeutic response. These findings are compatible with a pathway-based interpretation in which downstream inflammatory mediators may provide useful candidate signals.

Importantly, no soluble circulating biomarker identified in this review fulfills the analytic-validity, clinical-validity, and clinical-utility criteria required for routine implementation in psoriasis treatment selection. The existing evidence is limited by methodological heterogeneity, absence of prospective validation cohorts, lack of standardized assays and clinically meaningful cut-off values, insufficient cost-effectiveness analyses, and absence of evidence that biomarker-guided prescribing improves patient outcomes. These findings should therefore be interpreted as hypothesis-generating rather than clinically actionable. At present, soluble circulating biomarker-guided treatment selection in psoriasis cannot be recommended outside a research setting.

Future studies should prioritize prospective validation of pathway-based biomarker panels, particularly those including BD-2 and IL-17 isoforms, using standardized assays and predefined response endpoints. Until then, circulating biomarkers should be regarded as promising biological hypotheses rather than decision-making tools.

### Limitations

This study has several limitations. The most important limitation is the substantial clinical and methodological heterogeneity across included studies, including differences in treatment classes, outcome definitions (PASI75 vs. PASI90 vs. PASI100), and timing of response assessment. Although an exploratory pooling strategy was applied, this approach inherently reduces clinical interpretability and should be viewed as a trade-off to identify broader biological trends. Additional limitations include uneven data availability across therapeutic classes, small sample sizes for several biomarkers, and variability in biomarker measurement methods and reporting standards, which collectively restrict the robustness and generalizability of the findings. Many studies also lacked quantitative biomarker data or presented results in formats unsuitable for meta-analysis, necessitating an exploratory pooling approach in some cases. The limited number of studies reflects the scarcity of available biomarker datasets rather than methodological selection.

In addition, potential publication bias and selective reporting cannot be excluded, particularly given the predominance of small studies and subgroup analyses. A specific limitation concerns the SMD meta-analysis for IL-6, which was based on only two studies and a small total sample size. Accordingly, this analysis cannot provide a precise or robust estimate of a population-level effect. The wide confidence interval around the pooled SMD indicates substantial uncertainty regarding whether IL-6 levels meaningfully differ between responders and non-responders. In addition, with only two studies, estimation of between-study variance and heterogeneity is inherently unstable; therefore, the observed I^2^ value should not be overinterpreted. These findings should be considered evidence of insufficient available data rather than evidence for or against a clinically meaningful predictive effect of IL-6.

A further limitation is that the pooled *p*-value synthesis combines studies evaluating therapies with distinct mechanisms of action, different PASI response thresholds, and assessment timepoints ranging from early induction to long-term follow-up. As a result, the pooled z-scores should not be interpreted as treatment-specific predictive estimates, nor as evidence that a given biomarker has the same biological meaning across IL-17, IL-23, TNF, and TYK2 inhibition. A related limitation concerns the weighted Stouffer z-score synthesis. Because this method gives greater weight to larger studies, pooled z-scores may be driven partly by sample size rather than effect magnitude. Moreover, studies with non-concordant or non-conforming directions could not be integrated into a single directional pooled estimate without imposing assumptions about biological equivalence across therapies; therefore, the pooled *p*-value synthesis may overrepresent biomarkers with directionally concordant published signals. Thus, strong pooled signals, such as for BD-2, should not be interpreted as large or clinically meaningful effects. In addition, no formal correction for multiple testing was applied across biomarkers, so nominally significant findings should be considered exploratory. Finally, because few studies contributed to each biomarker analysis, Cochran’s Q and I^2^ had limited interpretability; absence of significant heterogeneity should not be taken as evidence of homogeneity.

## Figures and Tables

**Table 1:** Circulating biomarkers associated with systemic treatment response in psoriasis: summary of evidence.

IL-23/Th17 Pathway Biomarkers						
Biomarker	Position in Pathway	Direction in Responders	Studies with Association/Total Evaluated	Study Participants in Studies with Association/Total Number of Evaluated Study Participants	Therapeutic Class(es) Showing Association	Evidence Quality
IL-17A	Downstream effector	↑ Baseline → better response	3/7	667/2248	TYK2i (deucravacitinib); IL-23i (guselkumab)	Low—subgroup-specific ^a,b^; whole-cohort signal only with TYK2i [10,11,12]
IL-17F	Downstream effector	↑ Baseline → better response	1/4	344/1264	IL-23i (guselkumab)	Low—biologic-naive subgroup only [12]
IL-22	Downstream effector	↑ Baseline → better response	1/6	344/1373	IL-23i (guselkumab)	Low—TNFi-inadequate responder subgroup only [12]
TNF-α	Upstream/downstream	↓ Baseline → better response	1/6	344/808	IL-23i (guselkumab)	Low—TNFi-inadequate responder subgroup only [12]
IFN-γ	Downstream effector	~ Part of multi-marker signature	1/4	266/409	TNFi (etanercept; multi-marker model)	Low—no independent predictive signal; part of multi-marker signature only [13]
BD-2	Downstream effector (keratinocyte output)	↑ Baseline → better response	4/6	859/1093	IL-23i (guselkumab); IL-17i (secukinumab); TYK2i (deucravacitinib)	Moderate—Promising candidate—largest exploratory pooled z-score in *p*-value screening (z = 4.63, *p* < 0.001), but mixed evidence across studies and not an effect-size estimate; requires prospective validation [10,12,14,15].
**Systemic Inflammatory Biomarkers**						
**Biomarker**	**Category**	**Direction in Responders**	**Studies with Association/Total Evaluated**	**Study Participants in Studies with Association/Total Number of Evaluated Study Participants**	**Therapeutic Class(es) Showing Association**	**Evidence Quality**
IL-6	Pleiotropic cytokine	↓ Baseline → better response	1/6	344/808	IL-23i (guselkumab)	Low—biologic-naive subgroup only [12]
IL-19	Keratinocyte-derived IL	↑ Baseline → better response	1/1	203/203	TYK2i (deucravacitinib)	Preliminary—single study [10]
IL-13	Th2 cytokine	↑ Baseline → better response	1/2	28/62	IL-17i (secukinumab)	Preliminary—single study [16]
IL-18	Pro-inflammatory cytokine	↓ Baseline → better response	1/1	28/28	IL-17i (secukinumab)	Preliminary—single study; inverse to IL-13 [16]
IL-18/IL-13 ratio	Cytokine ratio	↓ Baseline ratio → better response	1/1	28/28	IL-17i (secukinumab)	Preliminary—single small study; AUC 0.86 for complete clearance (PASI = 0) at week 12 [16]
IL-17/IL-13 ratio	Cytokine ratio	↓ Baseline ratio → better response	1/1	28/28	IL-17i (secukinumab)	Preliminary—single small study; AUC 0.89 for complete clearance (PASI = 0) at week 12 [16]
IL-12	Pro-inflammatory cytokine	↑ Baseline → better response	1/2	43/71	TNFi (etanercept)	Preliminary—trend only; lost significance in multivariable model [17]
IL-12 receptor	Cytokine receptor	Association, not otherwise specified	1/1	266/266	TNFi (etanercept)	Preliminary—single study [13]
C-reactive protein (CRP)	Acute-phase reactant	↑ Baseline → better response	1/4	203/688	TYK2i (deucravacitinib)	Preliminary—association with deucravacitinib [10]; no association in 3 other studies
Serum calprotectin	Neutrophil-derived protein	↑ Baseline → better response	1/1	30/30	Conventional (methotrexate)	Preliminary—single study; PASI50 at month 3 [18]
CCL22/MDC	Chemokine	~ Association (direction NOS)	1/2	522/556	IL-23i (guselkumab)	Preliminary—direction of association not specified [19]
CCL20/CXCL10/GAL/LEP	Multi-protein inflammatory/cardiometabolic signature	↓ Baseline → better response	1/1	266/266	TNFi (etanercept; multi-marker model)	Preliminary—downregulated in responders as part of integrated protein signature [13]
**Hematologic Inflammatory Indices**						
**Biomarker**	**Category**	**Direction in Responders**	**Studies with Association/Total Evaluated**	**Study Participants in Studies with Association/Total Number of Evaluated Study Participants**	**Therapeutic Class(es) Showing Association**	**Evidence Quality**
SIRI	Composite hematologic index	↑ Baseline → better response	1/2	240/404	Multiple biologics + conventional	Preliminary—PASI100 at 6 months; no association for treatment persistence [20]
SII	Composite hematologic index	↑ Baseline → non-response/biologic switching	2/2	262/262	Multiple biologics	Low—predictor of biologic switching in 2 studies [21,22]; conflicting outcome definitions
NLR	Composite hematologic index	↑ Baseline → non-response	1/2	98/262	Multiple biologics	Preliminary—association in one study [22] only; no association for treatment persistence [21]
PLR	Composite hematologic index	↑ Baseline → non-response	2/2	262/262	Multiple biologics + conventional	Low—consistent signal across 2 studies for treatment discontinuation/switching [21,22]
d-NLR	Composite hematologic index	↑ Baseline → better response	1/1	240/240	Multiple biologics + conventional	Preliminary—CI crosses unity for PASI100; cautious interpretation [20]
**Cellular/Immunophenotypic Biomarkers**						
**Biomarker**	**Category**	**Direction in Responders**	**Studies with Association/Total Evaluated**	**Study Participants in Studies with Association/Total Number of Evaluated Study Participants**	**Therapeutic Class(es) Showing Association**	**Evidence Quality**
Neutrophil count/Platelet count	Routine hematologic markers	↑ Baseline → non-response (conventional)	1/1	164/164	Conventional systemics	Preliminary—no association for biologic retention [21]
Regulatory T cells (Tregs)	T-cell subset	↑ Baseline → non-response	1/1	14/14	TNFi (infliximab)	Preliminary—inverse association; TIGIT co-expression [23]
TIGIT on CD4^+^ T cells	Co-inhibitory receptor	↑ Baseline → non-response	1/1	14/14	TNFi (infliximab)	Preliminary—single study [23]
CD147 on CD4^+^ T/Th17 cells	T-cell surface marker	↓ Baseline → better response; ↑ Early change (wk 4) → better response	1/1	64/64	IL-17i (ixekizumab/secukinumab)	Preliminary—novel biomarker; also predicts relapse risk [24]
CD4^+^CD41a^+^ cells/Treg CD41a^+^	T-cell/platelet complex	↑ Baseline → better response	1/1	22/22	TNFi (adalimumab)	Preliminary—single study [25]
Monocyte doublets CD14 ^+^ CD16^+^ (≥0.4%)	Innate immune cell subset	↑ Baseline → better response	1/1	28/28	PDE4i (apremilast)	Preliminary—also identifies distinct gene expression profile in responders [26]
κ and λ free light chains	Humoral immune marker	↑ Baseline and post-treatment → better response	1/1	15/15	TNFi (adalimumab)	Preliminary—dynamic change also significant; single study [27]
**Metabolic Biomarkers**						
**Biomarker**	**Category**	**Direction in Responders**	**Studies with Association/Total Evaluated**	**Study Participants in Studies with Association/Total Number of Evaluated Study Participants**	**Therapeutic Class(es) Showing Association**	**Evidence Quality**
Triglycerides	Metabolic lipid	↓ Baseline → better response	1/1	66/66	IL-17i + IL-23i (pooled)	Preliminary—whole cohort PASI75/90; no subgroup signal [28]
Total cholesterol	Metabolic lipid	↓ Baseline → better response	1/1	66/66	IL-17i + IL-23i (pooled)	Preliminary—PASI90 only [28]
Leptin	Adipokine	↓ Baseline → better response (PASI90); ↑ Baseline → non-response (IL-23i subgroup)	1/1	66/66	IL-17i + IL-23i (pooled)	Preliminary—bidirectional signal by drug class subgroup [28]
ADMA	Endothelial dysfunction marker	↑ Baseline → better response	1/1	29/29	TNFi (anti-TNF-α therapy)	Preliminary—correlates with BSA and PASI reduction at 6 months [29]

Direction indicates the association between baseline biomarker levels and subsequent treatment response, unless otherwise specified. ↑: higher levels associated with response; ↓: lower levels associated with response; ~: direction not otherwise specified or part of a multi-marker composite; —: no association detected in any study. Studies with association/total evaluated: number of individual studies (not entries) reporting at least one statistically significant predictive association for that biomarker/total number of independent studies evaluating that biomarker for prediction of response. Evidence quality was qualitatively classified as: Moderate = signal reproduced in ≥2 independent studies and/or statistically significant in pooled analysis; Low = inconsistent or subgroup-restricted findings in ≥2 studies; Preliminary = evidence limited to a single study. For the participant-count metric (participants in studies reporting an association/total participants evaluated), samples were not subdivided according to the specific subgroups in which an association was observed; instead, the total number of participants enrolled in each study reporting an association was included. ^a^ IL-17A showed a whole-cohort signal only under deucravacitinib (TYK2 inhibitor); associations under guselkumab were restricted to biologic-naive or TNFi-inadequate-responder subgroups. ^b^ Subgroup specificity may reflect biological heterogeneity of the psoriasis population (biologic-naive vs. TNFi-experienced) rather than a true absence of effect. ADMA: asymmetric dimethylarginine; BD-2: β-defensin-2; CCL: C-C motif chemokine ligand; CRP: C-reactive protein; CXCL: C-X-C motif chemokine ligand; d-NLR: derived neutrophil-to-lymphocyte ratio; DNA: deoxyribonucleic acid; GAL: galectin; IFN: interferon; IL: interleukin; LEP: leptin (protein); MDC: macrophage-derived chemokine; NLR: neutrophil-to-lymphocyte ratio; NOS: not otherwise specified; PDE4i: phosphodiesterase 4 inhibitor; PLR: platelet-to-lymphocyte ratio; Ref: reference; SIRI: systemic inflammation response index; SII: systemic immune-inflammation index; TIGIT: T-cell immunoreceptor with Ig and ITIM domains; TNFi: tumour necrosis factor inhibitor; Treg: regulatory T cell; TYK2i: tyrosine kinase 2 inhibitor; IL-17i: interleukin-17 inhibitor; IL-23i: interleukin-23 inhibitor. For detailed study-by-study data (drug, response endpoint, effect size), see Supplementary Table S1.

## Data Availability

The data presented in this study are available upon reasonable request from the corresponding author.

## Supplementary Materials

The following supporting information can be downloaded at: https://www.mdpi.com/article/10.3390/jcm15156096/s1, Supplementary Material S1: PRISMA 2020 checklists [36]; Supplementary Material S2: Search query; Table S1: Detailed characteristics of studies evaluating predictive biomarkers; Table S2: Characteristics of the studies included in the systematic review; Table S3: Biomarkers evaluated in the included studies for which no predictive association with treatment response was found, organized by biological category [10,11,12,13,14,15,16,17,18,19,20,21,22,23,24,25,26,27,28,29,30,31,32,33,34,35].
