# Supplementary material for: Potential Blood Biomarkers Predicting Treatment Response in Psoriasis: A Systematic Review and Meta-Analysis
Source: J Clin Med. 2026 Aug 5;15(15):6096. doi: 10.3390/jcm15156096 (PMC13466316; doi:10.3390/jcm15156096)
Supplement: Supplementary file 1 [file jcm-15-06096-s001.zip › Supplementary Material S2.pdf]

SUPPLEMENTARY MATERIAL S2

MEDLINE Search query:
Date restriction: Last 10 years
("psoria*"[Title/Abstract] AND ("marker"[Title/Abstract] OR "biomarker"[Title/Abstract] OR "calprotectin"[Title/Abstract]) AND ("biologic"[Title/Abstract] OR "adalimumab"[Title/Abstract] OR "infliximab"[Title/Abstract] OR "certolizumab"[Title/Abstract] OR "etanercept"[Title/Abstract] OR "ustekinumab"[Title/Abstract] OR "ixekizumab"[Title/Abstract] OR "secukinumab"[Title/Abstract] OR "brodalumab"[Title/Abstract] OR "bimekizumab"[Title/Abstract] OR "risankizumab"[Title/Abstract] OR "tildrakizumab"[Title/Abstract] OR "guselkumab"[Title/Abstract] OR "golimumab"[Title/Abstract] OR "spesolimab"[Title/Abstract] OR "biosimilar"[Title/Abstract] OR "tumor necrosis factor alpha inhibitor"[Title/Abstract] OR "interleukin 17 inhibitor"[Title/Abstract] OR "interleukin 23 inhibitor"[Title/Abstract] OR "interleukin 12 23 inhibitor"[Title/Abstract] OR (("interleukine"[All Fields] OR "interleukines"[All Fields] OR "interleukins"[Supplementary Concept] OR "interleukins"[All Fields] OR "interleukin"[All Fields] OR "interleukins"[MeSH Terms]) AND "36 inhibitor"[Title/Abstract]) OR "tnf alpha inhibitor"[Title/Abstract] OR "il inhibitor"[Title/Abstract] OR "il 17 inhibitor"[Title/Abstract] OR "il 23 inhibitor"[Title/Abstract] OR "il 12 23 inhibitor"[Title/Abstract] OR "il 36 inhibitor"[Title/Abstract] OR "methotrexate"[Title/Abstract])) AND (y_10[Filter])

Web of Science Search query
Date restriction: Last 10 years
((psoria*) AND (((marker) OR (biomarker)) OR (calprotectin))) AND ((((((((((((((((((((((((((((biologic) OR (adalimumab)) OR (infliximab)) OR (certolizumab)) OR (etanercept)) OR (ustekinumab)) OR (ixekizumab)) OR (secukinumab)) OR (brodalumab)) OR (bimekizumab)) OR (rizankizumab)) OR (tildrakizumab)) OR (guselkumab)) OR (golimumab)) OR (spesolimab)) OR (biosimilar)) OR (tumor necrosis factor alpha inhibitor)) OR (interleukin 17 inhibitor)) OR (interleukin 23 inhibitor)) OR (interleukin 12/23 inhibitor)) OR (interleukin 36 inhibitor)) OR (TNF alpha inhibitor)) OR (IL inhibitor)) OR (IL 17 inhibitor)) OR (IL 23 inhibitor)) OR (IL 12/23 inhibitor)) OR (IL 36 inhibitor)) OR (methotrexate))
