# Supplementary material for: Potential Blood Biomarkers Predicting Treatment Response in Psoriasis: A Systematic Review and Meta-Analysis
Source: J Clin Med. 2026 Aug 5;15(15):6096. doi: 10.3390/jcm15156096 (PMC13466316; doi:10.3390/jcm15156096)
Supplement: Supplementary file 1 [file jcm-15-06096-s001.zip › Supplementary Tables.pdf]

SUPPLEMENTARY MATERIAL

Supplementary Table S1. Detailed characteristics of studies evaluating predictive biomarkers

IL-23 / Th17 pathway biomarkers					
**Biomarker***	**Study**	**Drug**	**Cutaneous response criteria**	**Biomarker status**	**Comments**
IL-23					
	[35]	Infliximab, certolizumab, adalimumab, secukinumab, ixekizumab	PASI90 (timepoint not specified)	No association	
	[19]	Guselkumab, placebo	PASI90 at week 48	No association	
	[19]	Guselkumab, placebo	PASI100 at week 28	No association	
	[16]	Secukinumab	PASI100 at week 12<br>PASI improvement	No association	
	[17]	Etanercept	PASI75 at week 24	No association	
IL-17A					
	[11]	Guselkumab	PASI100 at weeks 20 and 28	NR: higher (p-value N/A)	Only in patients with psoriasis >2 years; no association for the whole cohort
	[12]	Guselkumab	IGA 0/1 at week 24	R: higher (p=0.035)	Association found for previously inadequate responders to TNFi. No association for Biologic-naive patients nor for PASI75 or PASI90 at week 24 achievement
	[10]	Deucravacitinib, placebo	PASI75 at week 16	R: higher (p=0.02)<br>R: higher (p<0.05)	Association found for 6 and 12 mg groups
	[10]	Deucravacitinib, placebo	PASI improvement	R: high [OR 15.76, 95% CI 4.27–58.15)]	Association found for 6+12 mg analyzed together
	[19]	Guselkumab, placebo	PASI90 at week 48	No association	
	[19]	Guselkumab, placebo	PASI100 at week 28	No association	
	[35]	Infliximab, certolizumab, adalimumab, secukinumab, ixekizumab	PASI90 (timepoint not specified)	No association	
	[14]	Guselkumab, placebo	PASI improvement at week 100	Changes from baseline to week 24 predicted responder status (rho=0.41)	

	[17]	Etanercept	PASI75 at week 24	No association	
	[30]	Etanercept	PASI75 at week 12	No association	Pooled analysis including patients under treatment with etanercept, tofacitinib and placebo
	[15]	Secukinumab	PASI75 at week 16	Predictive data not reported	
IL-17F					
	[12]	Guselkumab	PASI75 at week 24	R: higher (p=0.018)	Association found for Biologic-naive patients. No association found for previously inadequate responders to TNFi nor for IGA 0/1 achievement
	[12]	Guselkumab	PASI90 at week 24	R: higher (p=0.008)	Association found for Biologic-naive patients. No association found for previously inadequate responders to TNFi nor for IGA 0/1 achievement
	[11]	Guselkumab	PASI100 at weeks 20 and 28	No association	
	[19]	Guselkumab, placebo	PASI90 at week 48	No association	
	[19]	Guselkumab, placebo	PASI100 at week 28	No association	
	[14]	Guselkumab, placebo	PASI improvement at week 100	Changes from baseline to week 24 predicted responder status (rho=0.41)	
IL-22					
	[12]	Guselkumab	PASI90 at week 24	R: higher (p=0.025)	Association found for previously inadequate responders to TNFi. No association for Biologic-naive patients nor for PASI90 or IGA 0/1 achievement at week 24
	[11]	Guselkumab	PASI100 at weeks 20 and 28	No association	
	[19]	Guselkumab, placebo	PASI90 at week 48	No association	
	[19]	Guselkumab, placebo	PASI100 at week 28	No association	
	[35]	Infliximab, certolizumab, adalimumab, secukinumab, ixekizumab	PASI90 (timepoint not specified)	No association	
	[14]	Guselkumab, placebo	PASI improvement at week 100	Changes from baseline to week 24 predicted responder status (rho=0.38)	
	[16]	Secukinumab	PASI100 at week 12	No association	

			PASI improvement		
TNF-α					
	[12]	Guselkumab	IGA 0/1 at week 24	R: lower (p=0.016)	Association found for previously inadequate responders to TNFi. No association for Biologic-naive patients nor for PASI75 or PASI90 achievement at week 24
	[35]	Infliximab, certolizumab, adalimumab, secukinumab, ixekizumab	PASI90 (timepoint not specified)	No association	
	[14]	Guselkumab, placebo	PASI improvement at week 100	No association	
	[16]	Secukinumab	PASI100 at week 12<br>PASI improvement	No association	
	[17]	Etanercept	PASI75 at week 24	No association	
	[15]	Secukinumab	PASI75 at week 16	Predictive data not reported	
IFN-γ					
	[16]	Secukinumab	PASI100 at week 12<br>PASI improvement	No association	
	[35]	Infliximab, certolizumab, adalimumab, secukinumab, ixekizumab	PASI90 (timepoint not specified)	No association	
	[13]	Etanercept	PASI75 at week 12	Association, not otherwise specified	Etanercept requires a combination of both inflammatory and cardiovascular disease biomarkers.
	[15]	Secukinumab	PASI75 at week 16	Predictive data not reported	
BD-2					
	[34]	Secukinumab	PASI75 at week 16	No association	
	[12]	Guselkumab	PASI90 at week 24	R: higher (p=0.013)<br>R: higher (p=0.004)	Association found for Biologic-naive and previously inadequate responders to TNFi patients, respectively. No association found for PASI75 achievement at week 24
	[12]	Guselkumab	IGA 0/1 at week 24	R: higher (p=0.005)	Association found for previously inadequate responders to TNFi. No association found for Biologic-naive patients nor

					for PASI75 achievement at week 24
	11	Guselkumab	PASI100 at weeks 20 and 28	No association	
	14	Guselkumab, placebo	PASI improvement at week 100	Changes from baseline to week 24 predicted responder status (rho=0.6)	
	10	Deucravacitinib	PASI improvement	R: High [OR 15.41 (95% CI 3.36–70.71)]	Association found for 6+12 mg analyzed together. No association for PASI75 at week 16, nor for deucravacitinib 6 mg and 12 mg groups analyzed separately
	15	Secukinumab	PASI75 at week 16	R: reduction at week 2	A rapid decrease in serum BD-2 at week 2 preceded PASI improvement at week 16; patients with early BD-2 reduction improved clinically, while the patient with persistently elevated BD-2 did not
**Systemic inflammatory biomarkers**					
**Biomarker***	**Study**	**Drug**	**Cutaneous response criteria**	**Biomarker status**	**Comments**
IL-6					
	12	Guselkumab	IGA 0/1 at week 24	R: lower (p=0.013)	Association found for Biologic-naive patients. No association found for previously inadequate responders to TNFi nor for PASI75 or PASI90 achievement at week 24
	35	Infliximab, certolizumab, adalimumab, secukinumab, ixekizumab	PASI90 (timepoint not specified)	No association	
	14	Guselkumab, placebo	PASI improvement at week 100	No association	
	16	Secukinumab	PASI100 at week 12<br>PASI improvement	No association	
	17	Etanercept	PASI75 at week 24	No association	
	15	Secukinumab	PASI75 at week 16	Predictive data not reported	
IL-19					
	10	Deucravacitinib, placebo	PASI75 at week 16	R: higher (p=0.02)	Association found only for

					Deucravacitinib 12 mg, not for 6 mg, or 6+12 mg group
	[10]	Deucravacitinib, placebo	PASI improvement	No association	No association for Deucravacitinib 6 mg, 12 mg, or 6+12 mg group
IL-13					
	[16]	Secukinumab	PASI100 at week 12	R: higher (p=0.002)	Strong positive (+20.23) predictor; cut-off 0.75 pg/mL, OR 28.0 (95% CI: 2.07–379.27)
	[15]	Secukinumab	PASI75 at week 16	Predictive data not reported	
IL-18					
	[16]	Secukinumab	PASI100 at week 12	R: lower (p=0.007)	Strong negative (–31.13) predictor; cut-off 0.4 pg/mL
IL-8					
	[19]	Guselkumab, placebo	PASI90 at week 48	No association	
	[30]	Etanercept	PASI75 at week 12	No association	
	[15]	Secukinumab	PASI75 at week 16	No association	
IL-18/IL-13 ratio					
	[16]	Secukinumab	PASI100 at week 12	R: lower (p=0.00001)	AUC 0.86, cut-off 0.5
	[16]	Secukinumab	PASI improvement	R: Moderate correlation (rho=-0.25)	
IL-17/IL-13					
	[16]	Secukinumab	PASI100 at week 12	R: lower (p=0.0044)	AUC 0.89, cut-off 0.44
	[16]	Secukinumab	PASI improvement	R: Moderate correlation (rho=-0.13)	
IL-12					
	[17]	Etanercept	PASI75 at week 24	R: higher (p=0.028)	
	[16]	Secukinumab	PASI100 at week 12<br>PASI improvement	No association	
IL-12 receptor					
	[13]	Etanercept	PASI75 at week 12	Association, not otherwise specified	Etanercept requires a combination of both inflammatory and cardiovascular disease biomarkers.
IL-1α					
	[16]	Secukinumab	PASI100 at week 12<br>PASI improvement	No association	
	[15]	Secukinumab	PASI75 at week 16	No association	
IL-1ß					
	[16]	Secukinumab	PASI100 at week 12	No association	

			PASI improvement		
	15	Secukinumab	PASI75 at week 16	No association	
IL-2					
	16	Secukinumab	PASI100 at week 12<br>PASI improvement	No association	
	15	Secukinumab	PASI75 at week 16	No association	
IL-4					
	16	Secukinumab	PASI100 at week 12<br>PASI improvement	No association	
	15	Secukinumab	PASI75 at week 16	No association	
IL-5					
	16	Secukinumab	PASI100 at week 12<br>PASI improvement	No association	
	15	Secukinumab	PASI75 at week 16	No association	
IL-10					
	16	Secukinumab	PASI100 at week 12<br>PASI improvement	No association	
	15	Secukinumab	PASI75 at week 16	No association	
IL-15					
	16	Secukinumab	PASI100 at week 12<br>PASI improvement	No association	
	15	Secukinumab	PASI75 at week 16	No association	
CRP					
	14	Guselkumab, placebo	PASI improvement at week 100	No association	
	10	Deucravacitinib	PASI75 at week 16	R: High [OR 5.40 (95% CI 1.76–16.58)]<br>R: Low [OR 2.84 (95% CI 1.00–8.11)]	Association found for 6+12 mg analyzed together. No association for deucravacitinib 6 mg and 12 mg groups analyzed separately
	17	Etanercept	PASI75 at week 24	No association	
	21	Biologics: n=51 (Infliximab, Adalimumab, Secukinumab, Ixekizumab, Brodalumab, Guselkumab, Tildrakizumab, Risankizumab)<br><br>Conventional systemics: n=48 (apremilast, methotrexate, cyclosporine, etretinate)	Treatment continuation rate (persistence) at 1 year	No association	
Serum calprotectin					
	18	Methotrexate	PASI50 at month 3	R: higher (p=0.015)	Cut-off 60.0 ng/mL, AUC

					0.762 (p=0.015; 95% CI 0.577–0.948)
CCL22/MDC					
	[19]	Guselkumab, placebo	PASI90 at week 48	R: association, not otherwise specified (p-value N/A)	No association for PASI100 at week 28
	[15]	Secukinumab	PASI75 at week 16	No association	
CXCL10					
	[13]	Etanercept	PASI75 at week 12	Association, not otherwise specified	Etanercept requires a combination of both inflammatory and cardiovascular disease biomarkers.
CCL20					
	[13]	Etanercept	PASI75 at week 12	Association, not otherwise specified	Etanercept requires a combination of both inflammatory and cardiovascular disease biomarkers.
GAL					
	[13]	Etanercept	PASI75 at week 12	R: lower (p-value N/A)	Etanercept requires a combination of both inflammatory and cardiovascular disease biomarkers.
LEP					
	[13]			R: lower (p-value N/A)	Etanercept requires a combination of both inflammatory and cardiovascular disease biomarkers.
MIP-1β					
	[19]	Guselkumab, placebo	PASI90 at week 48	No association	
	[30]	Etanercept	PASI75 at week 12	No association	
	[15]	Secukinumab	PASI75 at week 16	No association	
TARC					
	[30]	Etanercept	PASI75 at week 12	No association	
	[15]	Secukinumab	PASI75 at week 16	No association	
IP-10					
	[30]	Etanercept	PASI75 at week 12	No association	
	[15]	Secukinumab	PASI75 at week 16	No association	
**Hematologic inflammatory indices**					

Biomarker*	Study	Drug	Cutaneous response criteria	Biomarker status	Comments
SIRI					
	[20]	adalimumab, etanercept, Infliximab, certolizumab, ixekizumab, secukinumab, tildrakizumab, risankizumab, guselkumab, ustekinumab, apremilast	PASI100 at 6 months	R: higher (p=0.04)	
	[21]	Biologics: n=51 (Infliximab, Adalimumab, Secukinumab, Ixekizumab, Brodalumab, Guselkumab, Tildrakizumab, Risankizumab)<br><br>Conventional systemics: n=48 (apremilast, methotrexate, cyclosporine, etretinate)	Treatment continuation rate (persistence) at 1 year	No association	
SII					
	[22]	Adalimumab, ustekinumab, etanercept, secukinumab	Biologic maintenance	NR: higher (p<0.001)	
	[21]	Biologics: n=51 (Infliximab, Adalimumab, Secukinumab, Ixekizumab, Brodalumab, Guselkumab, Tildrakizumab, Risankizumab)<br><br>Conventional systemics: n=48 (apremilast, methotrexate, cyclosporine, etretinate)	Treatment continuation rate (persistence) at 1 year	NR: higher (p=0.0383)	Association only found for conventional systemic treatment, not biologics
NLR					
	[22]	Adalimumab, ustekinumab, etanercept, secukinumab	Biologic maintenance	NR: higher (p<0.001)	
	[21]	Biologics: n=51 (Infliximab, Adalimumab, Secukinumab, Ixekizumab, Brodalumab, Guselkumab, Tildrakizumab, Risankizumab)<br><br>Conventional systemics: n=48 (apremilast, methotrexate, cyclosporine, etretinate)	Treatment continuation rate (persistence) at 1 year	No association	
PLR					
	[22]	Adalimumab, ustekinumab, etanercept, secukinumab	Biologic maintenance	NR: higher (p<0.001)	
	[21]	Biologics: n=51 (Infliximab,	Treatment continuation rate	NR: higher (p=0.0107)	Association only found for

		Adalimumab, Secukinumab, Ixekizumab, Brodalumab, Guselkumab, Tildrakizumab, Risankizumab)<br><br>Conventional systemics: n=48 (apremilast, methotrexate, cyclosporine, etretinate)	(persistence) at 1 year		conventional systemic treatment, not biologics
d-NLR					
	[20]	adalimumab, etanercept, Infliximab, certolizumab, ixekizumab, secukinumab, tildrakizumab, risankizumab, guselkumab, ustekinumab, apremilast	PASI100 at 6 months	R: higher (p=0.05)	
**Collagen-related biomarkers**					
**Biomarker***	**Study**	**Drug**	**Cutaneous response criteria**	**Biomarker status**	**Comments**
C1M					
	[14]	Guselkumab, placebo	PASI improvement at week 100	No association	
	[31]	Guselkumab, placebo	PASI75 at week 24	No association	
C3M					
	[14]	Guselkumab, placebo	PASI improvement at week 100	No association	
	[31]	Guselkumab, placebo	PASI75 at week 24	No association	
C4M					
	[14]	Guselkumab, placebo	PASI improvement at week 100	No association	
	[31]	Guselkumab, placebo	PASI75 at week 24	No association	
C6M					
	[14]	Guselkumab, placebo	PASI improvement at week 100	No association	
	[31]	Guselkumab, placebo	PASI75 at week 24	No association	
**Cellular / immunophenotypic biomarkers**					
**Biomarker***	**Study**	**Drug**	**Cutaneous response criteria**	**Biomarker status**	**Comments**
Neutrophil count					
	[21]	Biologics: n=51 (Infliximab, Adalimumab, Secukinumab, Ixekizumab, Brodalumab, Guselkumab, Tildrakizumab, Risankizumab)<br><br>Conventional systemics: n=48 (apremilast, methotrexate, cyclosporine, etretinate)	Treatment continuation rate (persistence) at 1 year	NR: higher (p<0.0001)	Association only found for conventional systemic treatment, not biologics

Platelet count					
	[21]	Biologics: n=51 (Infliximab, Adalimumab, Secukinumab, Ixekizumab, Brodalumab, Guselkumab, Tildrakizumab, Risankizumab)<br><br>Conventional systemics: n=48 (apremilast, methotrexate, cyclosporine, etretinate)	Treatment continuation rate (persistence) at 1 year	NR: higher (p=0.0006)	Association only found for conventional systemic treatment, not biologics
Regulatory T cells					
	[23]	Infliximab	PASI improvement at week 10	NR: higher (p=0.0127)	
TIGIT					
	[23]	Infliximab	PASI improvement at week 10	NR: higher (p=0.0435)	
CD147 expression on $CD4^+$ T and Th17 cells					
	[24]	Ixekizumab, secukinumab	PASI improvement	R: lower (p-value N/A)	Positive correlation between CD147 expression after 4 weeks
$CD4^+CD41a^+$ and Treg $CD41a^+$					
	[25]	Adalimumab	PASI75 at week 24	R: higher (p<0.05)	
Monocyte doublets $CD14^+CD16^+$					
	[26]	Apremilast	PASI50, PASI75 and PASI reduction at week 16	R: >0.4 (p=0.035)	
κ and λ free light chains					
	[27]	Adalimumab	PASI75 (timepoint not specified)	R: higher (p-value N/A)	
**Metabolic biomarkers**					
**Biomarker***	**Study**	**Drug**	**Cutaneous response criteria**	**Biomarker status**	**Comments**
Triglycerides					
	[28]	Secukinumab, ixekizumab, bimekizumab, guselkumab, risankizumab, tildrakizumab	PASI75 at week 16	R: lower (p=0.037)	No association for IL-17 and IL-23 inhibitors separately analyzed
	[28]	Secukinumab, ixekizumab,	PASI90 at week 16	R: lower (p=0.018)	No association for IL-17 and

		bimekizumab, guselkumab, risankizumab, tildrakizumab			IL-23 inhibitors separately analyzed
Total cholesterol					
	[28]	Secukinumab, ixekizumab, bimekizumab, guselkumab, risankizumab, tildrakizumab	PASI90 at week 16	R: lower (p=0.047)	No association for PASI75 at week 16 nor for IL-17 and IL-23 inhibitors separately analyzed
Leptin					
	[28]	Secukinumab, ixekizumab, bimekizumab, guselkumab, risankizumab, tildrakizumab	PASI90 at week 16	R: lower (p=0.042)	No association for PASI75 at week 16 nor for IL-17 separately analyzed
	[28]	Guselkumab, risankizumab, tildrakizumab	PASI90 at week 16	NR: higher (p=0.016)	No association for PASI75 at week 16 nor for IL-17 separately analyzed
ADMA					
	[29]	Adalimumab	Change in BSA and PASI after 6 months	R: higher (p-value N/A)	

*Baseline biomarker level unless otherwise specified.

↑: higher levels associated with response; ↓: lower levels associated with response.

"Association not otherwise specified" indicates inclusion in multivariable or multi-marker models without independent predictive effect.

Abbreviations: ADMA: asymmetric dimethylarginine; BD-2: β-defensin-2; BSA: body surface area; CCL: C-C motif chemokine ligand; CRP: C-reactive protein; CXCL: C-X-C motif chemokine ligand; d-NLR: derived neutrophil-to-lymphocyte ratio; DNA: deoxyribonucleic acid; IGA: Investigator Global Assessment; IL: interleukin; IP-10: interferon-inducible protein-10; LEP: leptin (protein); MIP-1β: macrophage inflammatory protein-1β; NLR: neutrophil-to-lymphocyte ratio; NR: non-responder; PASI: Psoriasis Area and Severity Index; PLR: platelet-to-lymphocyte ratio; R: responder; SIRI: systemic inflammation response index; SII: systemic immune-inflammation index; TARC: thymus- and activation-regulated chemokine; TIGIT: T cell immunoreceptor with Ig and ITIM domains; TNFi: tumour necrosis factor inhibitor.

Supplementary Table S2. Characteristics of the studies included in the systematic review

Study	Criteria indicating low risk of bias	Type of study	n (M:F), age	Diagnosis	Drug n (M:F)*
Cardner[34]	4/8	Retrospective observational study of RCTs	114 (N/A)	Ps + PsA	Secukinumab
Morariu[20]	6/8	Retrospective observational study	240 (143:97)	Ps ± PsA	Adalimumab 22 (N/A)<br>Etanercept 18 (N/A)<br>Infliximab 6 (N/A)<br>Certolizumab 6 (N/A)<br>Ixekizumab 61 (N/A)<br>Secukinumab 41 (N/A)<br>Tildrakizumab 11 (N/A)<br>Risankizumab 44 (N/A)<br>Guselkumab 10 (N/A)<br>Ustekinumab 17 (N/A)<br>Apremilast 4 (N/A)
Larson[26]	5/8	Prospective observational study	28 (18:10)	Ps	Apremilast
Schäkel[11]	6/8	RCT subanalysis	120 (N/A)	Ps ± PsA	Guselkumab
Gordon[19]	7/8	Double-blinded controlled RCT	522 (N/A)	Ps	Guselkumab 375 (257:118)<br>Placebo 147 (N/A)
Sanz-Martinez[25]	5/8	Observational prospective study	22 (19:3)	Ps ± PsA	Adalimumab
Vandikas[33]	6/8	Observational prospective study	20 (13:7)	Ps ± PsA	Etanercept
Miyagawa[35]	5/8	Observational retrospective study	81 (N/A)	Ps + PsA	Cohort 1 (exploratory cohort) 47 (N/A): TNFi 24 (N/A) (infliximab 1, certolizumab pegol 1, adalimumab 22); IL-17 inhibitor 23 (N/A) (secukinumab 18, ixekizumab 5)<br><br>Cohort 2 (validation cohort) 34 (N/A): TNFi 20 (N/A); IL-17 inhibitor 14 (N/A)
Belli[28]	6/8	Observational prospective study	66 (N/A)	Ps ± PsA	Secukinumab<br>Ixekizumab Bimekizumab<br>Guselkumab Risankizumab<br>Tildrakizumab
Sofíudóttir[32]	3/8	RCT	10 (N/A)	Ps	Biologic NOS

Siebert[14]	5/8	RCT	278 (157:121)	Ps + PsA	Inflammatory biomarker cohort:<br>Guselkumab 100 (N/A)<br>Placebo 50 (N/A)<br><br>Collagen biomarker cohort:<br>Guselkumab 178 (N/A)
Siebert[12]	7/8	Compilation of various RCT	344 (166:178)	Ps + PsA	Guselkumab 237 (N/A)<br>Placebo 107 (N/A)T
FitzGerald[10]	6/8	RCT placebo controlled	203 (N/A)	Ps + PsA	Deucravacitinib 137 (N/A)<br>Placebo 66 (N/A)
Hamza[18]	5/8	Observational prospective study	30 (20:10)	Ps	MTX
Ziolkowska-Banasik[16]	5/8	Observational prospective study	28 (15:13)	Ps	Secukinumab
Liu[24]	4/8	Observational prospective study	64 (49:15)	Ps	Ixekizumab<br>Secukinumab
Lu[17]	5/8	Observational prospective study	43 (27:16)	Ps	Etanercept
Di Caprio[27]	3/8	Observational prospective study	15 (7:8)	Ps	Adalimumab
Schett[31]	5/8	Subset of RCT	260 (142/118)	Ps + PsA	Guselkumab 174 (100/74)<br>Placebo with crossover to guselkumab 86 (42/44)
Murray[22]	5/8	Observational retrospective study	98 (62:36)	Ps ± PsA	Adalimumab 60 (N/A)<br>Ustekinumab 20 (N/A)<br>Etanercept 17 (N/A)<br>Secukinumab 1 (N/A)
Tomalin[13]	7/8	Observational retrospective study from RCT	266 (184:82)	Ps	Etanercept 50 (89:39)
Fitz[31]	6/8	RCT	623 (N/A)	Ps	Etanercept 190 (N/A)<br>Placebo 60 (N/A)
Pina[29]	6/8	Observational prospective study	29 (14:15)	Ps	Adalimumab
Morita[15]	5/8	Observational prospective study	34 (N/A)	Ps	Secukinumab
Sugimoto[21]	5/8	Observational retrospective study	164 (109:55)	Ps ± PsA	Biologics: n=51 (Infliximab, Adalimumab, Secukinumab, Ixekizumab, Brodalumab, Guselkumab, Tildrakizumab, Risankizumab)<br><br>Conventional systemics: n=48 (apremilast, methotrexate, cyclosporine, etretinate)
Yu[23]	4/8	Observational prospective study	14 (11:3)	Ps	Infliximab

F: Female; M: Male; N/A: Not available; NOS: Not Otherwise Specified; Ps: Psoriasis; PsA: Psoriatic Arthritis; ROB: Risk of Bias

*Only specified if ≥1 drugs were used

Supplementary Table S3. Biomarkers evaluated in the included studies for which no predictive association with treatment response was found, organized by biological category

**IL-23/Th17 pathway cytokines**		
**Biomarker**	**Treatment class evaluated**	**Reference(s)**
IL-17 (not otherwise specified)	IL-17i (secukinumab)	[16]
IL-17A/F (combined)	TNFi (etanercept) + JAKi (tofacitinib)	[30]
IL-21	TNFi + IL-17i + IL-23i (mixed biologics)	[35]
IL-23	TNFi (etanercept); IL-17i (secukinumab); IL-23i (guselkumab); mixed biologics	[16,17,19,35]
**Pro-inflammatory cytokines (non-Th17)**		
**Biomarker**	**Treatment class evaluated**	**Reference(s)**
GM-CSF	IL-17i (secukinumab)	[15]
IFN-α	IL-17i (secukinumab)	[16]
IL-1α	IL-17i (secukinumab) × 2	[15,16]
IL-1β	IL-17i (secukinumab) × 2	[15,16]
IL-2	IL-17i (secukinumab) × 2	[15,16]
IL-4	IL-17i (secukinumab) × 2	[15,16]

IL-5	IL-17i (secukinumab) × 2	15,16
IL-7	IL-17i (secukinumab)	15
IL-8	IL-23i (guselkumab); TNFi (etanercept) + JAKi (tofacitinib); IL-17i (secukinumab)	15,19,30
IL-9	IL-17i (secukinumab)	16
IL-10	IL-17i (secukinumab) × 2	15,16
IL-12/IL-23p40	IL-17i (secukinumab)	15
IL-12p70	IL-17i (secukinumab)	15
IL-15	IL-17i (secukinumab) × 2	15,16
IL-16	IL-17i (secukinumab)	15
TNF-β	IL-17i (secukinumab)	15
**Chemokines and vascular mediators**		
**Biomarker**	**Treatment class evaluated**	**Reference(s)**
E-selectin	TNFi (etanercept) + JAKi (tofacitinib)	30
Eotaxin (CCL11)	IL-17i (secukinumab)	15
Eotaxin-3 (CCL26)	IL-17i (secukinumab)	15

IP-10 (CXCL10)	TNFi (etanercept) + JAKi (tofacitinib); IL-17i (secukinumab)	15,30
MCP-1 (CCL2)	IL-17i (secukinumab)	15
MCP-4 (CCL13)	IL-17i (secukinumab)	15
MIG (CXCL9)	TNFi (etanercept) + JAKi (tofacitinib)	30
MIP-1α (CCL3)	IL-17i (secukinumab)	15
MIP-1β (CCL4)	IL-23i (guselkumab); TNFi (etanercept) + JAKi (tofacitinib); IL-17i (secukinumab)	15,19,30
MIP-3β (CCL19)	TNFi (etanercept) + JAKi (tofacitinib)	30
TARC (CCL17)	TNFi (etanercept) + JAKi (tofacitinib); IL-17i (secukinumab)	15,30
VEGF-A	IL-17i (secukinumab)	15
**Collagen turnover biomarkers**		
**Biomarker**	**Treatment class evaluated**	**Reference(s)**
C1M (type I collagen degradation marker)	IL-23i (guselkumab) × 2	14,31
C2M (type II collagen degradation marker)	IL-23i (guselkumab)	31

C3M (type III collagen degradation marker)	IL-23i (guselkumab) × 2	14,31
C4M (type IV collagen degradation marker)	IL-23i (guselkumab) × 2	14,31
C6M (type VI collagen degradation marker)	IL-23i (guselkumab) × 2	14,31
C10C (type X collagen marker)	IL-23i (guselkumab)	31
PRO-C1 (type I collagen formation marker)	IL-23i (guselkumab)	31
PRO-C2 (type II collagen formation marker)	IL-23i (guselkumab)	31
PRO-C3 (type III collagen formation marker)	IL-23i (guselkumab)	31
PRO-C4 (type IV collagen formation marker)	IL-23i (guselkumab)	31
PRO-C6 (type VI collagen formation marker)	IL-23i (guselkumab)	31
**Acute-phase and non-specific inflammatory markers**		
**Biomarker**	**Treatment class evaluated**	**Reference(s)**
ESR (erythrocyte sedimentation rate)	TNFi (etanercept)	17
SAA (serum amyloid A)	IL-23i (guselkumab)	14

Cellular and immunophenotypic markers		
**Biomarker**	**Treatment class evaluated**	**Reference(s)**
Antinuclear antibodies	TNFi (adalimumab)	[27]
BTLA	TNFi (infliximab)	[23]
$CD4^{+}CD161^{+}CD41a^{+}$ cells	TNFi (adalimumab)	[25]
CIRs (co-inhibitory receptors; composite score)	TNFi (infliximab)	[23]
CTLA-4	TNFi (infliximab)	[23]
Immunoglobulins (total)	TNFi (adalimumab)	[27]
Intermediate monocytes	PDE4i (apremilast)	[26]
LAG-3	TNFi (infliximab)	[23]
Lymphocyte count	Multiple biologics + conventional	[21]
MLR (monocyte-to-lymphocyte ratio)	Multiple biologics + conventional	[21]
Monocyte count	Multiple biologics + conventional	[21]
Monocyte-platelet aggregates (MPAs)	PDE4i (apremilast)	[26]
PDPN (podoplanin)	TNFi (infliximab)	[23]

PROCR (endothelial protein C receptor)	TNFi (infliximab)	23
TIM-3	TNFi (infliximab)	23
**Metabolic and adipokine biomarkers**		
**Biomarker**	**Treatment class evaluated**	**Reference(s)**
Adipokines (general; not otherwise specified)	TNFi (anti-TNF-α therapy)	29
Adiponectin	IL-17i + IL-23i (combined cohort)	28
HDL cholesterol	IL-17i + IL-23i (combined cohort)	28
LDL cholesterol	IL-17i + IL-23i (combined cohort)	28
Osteoprotegerin (OPG)	TNFi (anti-TNF-α therapy)	29
Resistin	TNFi (anti-TNF-α therapy)	29
Total 25(OH)D [25(OH)D2 + 25(OH)D3] (vitamin D)	TNFi (etanercept)	33
Visfatin	IL-17i + IL-23i (combined cohort)	28
**Multi-protein and proteomics panels**		
**Biomarker**	**Treatment class evaluated**	**Reference(s)**

MFAP4 (microfibrillar-associated protein 4)	Multiple biologics (BELIEVE cohort)	32
Olink proximity extension assay panel: 92 inflammation-associated and 65 cardiovascular disease-associated proteins (individual-level analysis)†	TNFi (etanercept) + JAKi (tofacitinib)	13

Biomarkers listed represent those evaluated in their respective studies for prediction of cutaneous response to systemic therapy, for which no statistically significant predictive association was identified.

Where the same reference number appears multiple times (× 2), the biomarker was evaluated across two independent studies sharing the same therapeutic approach.

† The Olink panel was evaluated as individual proteins. While most of the 157 proteins showed no independent predictive association, a subset (CCL20, CXCL10, IFN-γ, IL-12 receptor B, GAL, LEP) was significantly associated with etanercept response as part of an integrated multi-protein signature.

BTLA: B and T lymphocyte attenuator; CCL: C-C motif chemokine ligand; CIRs: co-inhibitory receptors; CTLA-4: cytotoxic T-lymphocyte-associated protein 4; CXCL: C-X-C motif chemokine ligand; DNA: deoxyribonucleic acid; ESR: erythrocyte sedimentation rate; GAL: galectin; GM-CSF: granulocyte-macrophage colony-stimulating factor; HDL: high-density lipoprotein; IFN: interferon; IL: interleukin; IP-10: interferon-inducible protein-10 (CXCL10); JAKi: Janus kinase inhibitor; LAG-3: lymphocyte-activation gene 3; LDL: low-density lipoprotein; LEP: leptin (protein); MFAP4: microfibrillar-associated protein 4; MCP: monocyte chemoattractant protein; MIG: monokine induced by gamma interferon (CXCL9); MIP: macrophage inflammatory protein; MLR: monocyte-to-lymphocyte ratio; MPA: monocyte-platelet aggregate; NOS: not otherwise specified; PDPN: podoplanin; PDE4i: phosphodiesterase 4 inhibitor; PROCR: endothelial protein C receptor; PRO-C: collagen formation marker; SAA: serum amyloid A; TARC: thymus- and activation-regulated chemokine (CCL17); TIM-3: T-cell immunoglobulin and mucin domain-containing protein 3; TNF: tumour necrosis factor; TNFi: tumour necrosis factor inhibitor; VEGF: vascular endothelial growth factor; IL-17i: interleukin-17 inhibitor; IL-23i: interleukin-23 inhibitor; 25(OH)D: 25-hydroxyvitamin D.
